# Glycosylation in kidney diseases

**DOI:** 10.1093/pcmedi/pbaf017

**Published:** 2025-07-11

**Authors:** Yingying Ling, Fei Cai, Tao Su, Yi Zhong, Ling Li, Bo Meng, Guisen Li, Meng Gong, Hao Yang, Xinfang Xie, Zhenyu Sun, Yang Zhao, Fang Liu, Yong Zhang

**Affiliations:** Department of Nephrology, Institutes for Systems Genetics, Frontiers Science Center for Disease-related Molecular Network, West China Hospital, Sichuan University, Chengdu 610041, China; Department of Nephrology, Institutes for Systems Genetics, Frontiers Science Center for Disease-related Molecular Network, West China Hospital, Sichuan University, Chengdu 610041, China; Department of Nephrology, Institutes for Systems Genetics, Frontiers Science Center for Disease-related Molecular Network, West China Hospital, Sichuan University, Chengdu 610041, China; Department of Nephrology, Institutes for Systems Genetics, Frontiers Science Center for Disease-related Molecular Network, West China Hospital, Sichuan University, Chengdu 610041, China; Department of Nephrology, Institute of Kidney Diseases, West China Hospital, Sichuan University, Chengdu 610041, China; Technology Innovation Center of Mass Spectrometry for State Market Regulation, Center for Advanced Measurement Science, National Institute of Metrology, Beijing 100029, China; Renal Department and Institute of Nephrology, Sichuan Provincial People's Hospital, University of Electronic Science and Technology of China, Sichuan Clinical Research Center for Kidney Diseases, Chengdu 611731, China; Department of Nephrology, Institutes for Systems Genetics, Frontiers Science Center for Disease-related Molecular Network, West China Hospital, Sichuan University, Chengdu 610041, China; Transplant Center and NHC Key Lab of Transplant Engineering and Immunology, West China Hospital, Sichuan University, Chengdu 610041, China; Department of Nephrology, The First Affiliated Hospital of Xi'an Jiaotong University, Xi'an 710061, China; Department of Pathology, Johns Hopkins University, Baltimore, MD 21231, United States; Technology Innovation Center of Mass Spectrometry for State Market Regulation, Center for Advanced Measurement Science, National Institute of Metrology, Beijing 100029, China; Department of Nephrology, Institutes for Systems Genetics, Frontiers Science Center for Disease-related Molecular Network, West China Hospital, Sichuan University, Chengdu 610041, China; Department of Nephrology, Institutes for Systems Genetics, Frontiers Science Center for Disease-related Molecular Network, West China Hospital, Sichuan University, Chengdu 610041, China

**Keywords:** glycosylation, kidney diseases, glycoproteomics, mass spectrometry, biomarkers

## Abstract

Protein glycosylation is a critical post-translational modification that influences protein folding, localization, stability, and functional interactions by attaching glycans to specific sites. This process is crucial for biological functions of glycoproteins, and aberrant glycosylation can lead to genetic disorders, immune system issues, and multi-organ pathologies. Recent advancements in glycoproteomic technologies have made the study of protein glycosylation a key focus for understanding the pathogenesis of kidney diseases. This review provides a comprehensive overview of protein glycosylation mechanisms, its biological roles, molecular pathways, and significant functions in renal physiology and pathology. It specifically highlights the dynamic changes and regulatory networks associated with aberrant glycosylation in kidney diseases such as immunoglobulin A nephropathy, diabetic kidney disease, autosomal dominant polycystic kidney disease, renal cell carcinoma, and acute kidney injury. It also evaluates the clinical applications of related technologies and biomarkers. Additionally, it discusses the challenges in developing glycosylation-targeted therapeutic strategies. Future research should focus on clarifying cell-specific glycosylation regulatory networks in the kidney, integrating glycobiology with multi-omics approaches, and improving precision diagnostics and treatment for kidney diseases.

## Graphical Abstract

**Graphical Abstract:**
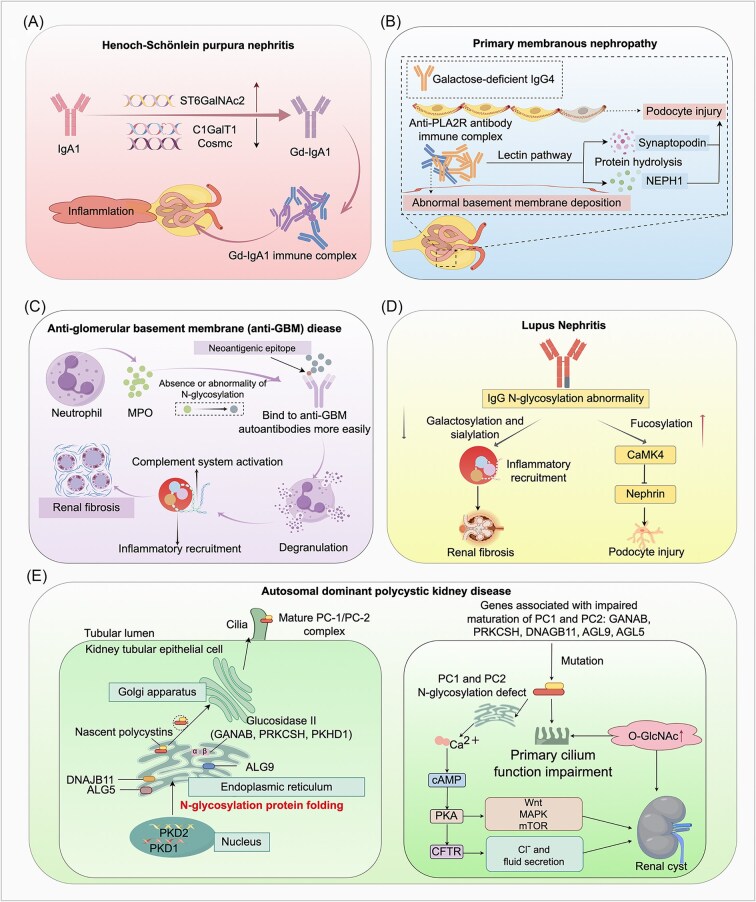
Schematic diagram of glycosylation in nephropathy pathogenesis.

## Introduction

The kidney is a vital organ that plays a crucial role in maintaining the homeostasis of the human body's internal milieu. It exquisitely orchestrates the regulation of water and osmotic pressure, safeguards acid–base balance, secretes bioactive molecules, and engages in intricate crosstalk with multiple organ systems to fulfill its physiological functions [1, 2]. However, in 2017, kidney diseases represent a critical global health challenge characterized by rising incidence and mortality rates. Alarmingly, the global prevalence of chronic kidney disease (CKD) reached a staggering 9.1%, affecting 700 million individuals across all disease stages [3]. Moreover, CKD was responsible for 1.2 million deaths, making it the 12th leading cause of mortality worldwide [3]. Acute kidney injury (AKI) represents a critical precursor to CKD development. Statistics from 2017 indicate that the global number of AKI patients approximated 13.3 million [4]. Clinical evidence shows that AKI is strongly linked to increased risks of severe short- and long-term complications, involving extra-renal organ systems and escalating healthcare costs [4]. Despite the growing global burden of kidney diseases, the effective treatments are profoundly limited [5, 6]. Therefore, comprehensively elucidating the molecular mechanisms driving kidney disease progression is essential for advancing diagnostics and therapies. In this context, multiple post-translational modifications (PTMs) critically regulate disease pathways, making the unraveling of their mechanisms key to the discovery of novel therapeutic targets [7].

Among >300 known PTMs, glycosylation stands out as one of the most ubiquitous and functionally critical modifications, occurring in >50% of human proteins [8, 9]. This process primarily occurs in the endoplasmic reticulum (ER) and Golgi apparatus, where structurally diverse oligosaccharide chains are covalently attached to specific amino acid residues through glycosidic linkages [10]. These glycan compositions and structures carry significant biological information [11]. Human glycosylation encompasses 16 distinct pathways, including 14 forms of protein glycosylation and two types of lipid glycosylation. Protein glycosylation is broadly classified into *N*-glycosylation, *O*-glycosylation, glycosylphosphatidylinositol (GPI) anchor linkage, tryptophan *C*-mannosylation, *S*-glycosylation (e.g. cysteine-*S*-glycosylation), and *P*-glycosylation (e.g. phosphorylation-associated glycosylation), with *N/O*-glycosylation representing the predominant subtypes [11].

Protein glycosylation regulates cellular functions through various pathways. Glycans have the capacity to modulate protein structure, subcellular localization, and trafficking, thereby exerting a profound impact on protein folding, activity, and stability. These effects, in turn, underpin fundamental biological processes such as cell–cell recognition, signal transduction, and immune responses [12, 13]. Recent advances in high-throughput glycoproteomic technology have enabled systematic analysis of glycoproteins in preclinical and clinical studies, revealing that aberrant glycosylation is closely associated with major diseases, including cancers, kidney disorders, neurodegenerative diseases, and metabolic conditions [12, 14–18]. Aberrant alterations in proteins and their attached glycans hold promise as diagnostic and prognostic biomarkers, and as therapeutic targets for managing or slowing disease progression. Therefore, understanding glycosylation modifications is essential for deciphering kidney disease mechanisms. In this review, we first briefly introduce the process of protein glycosylation, including its biological functions and underlying molecular mechanisms. We then summarize current glycomics analysis techniques with a particular focus on systematically elucidating the dynamic alterations in regulatory networks underlying aberrant glycosylation in major kidney diseases, including immunoglobulin A nephropathy (IgAN), diabetic kidney disease (DKD), autosomal dominant polycystic kidney disease (ADPKD), renal cell carcinoma (RCC), and AKI, etc. These representative diseases were selected for their significant global burden, distinct pathophysiologies, and the well-documented, critical roles that dysregulated protein glycosylation plays in their initiation, progression, and clinical manifestations. Furthermore, we discuss the potential of targeting glycosylation pathways and identifying novel diagnostic glycosylation biomarkers to develop novel therapeutic strategies. To advance this field, future research should aim to unravel kidney cell-specific glycosylation networks, integrate glycobiology with multi-omics approaches, and provide new perspectives for the precision diagnosis and targeted treatment of kidney diseases.

## Overview of protein glycosylation

### *N*-Glycosylation

**Figure 1.**
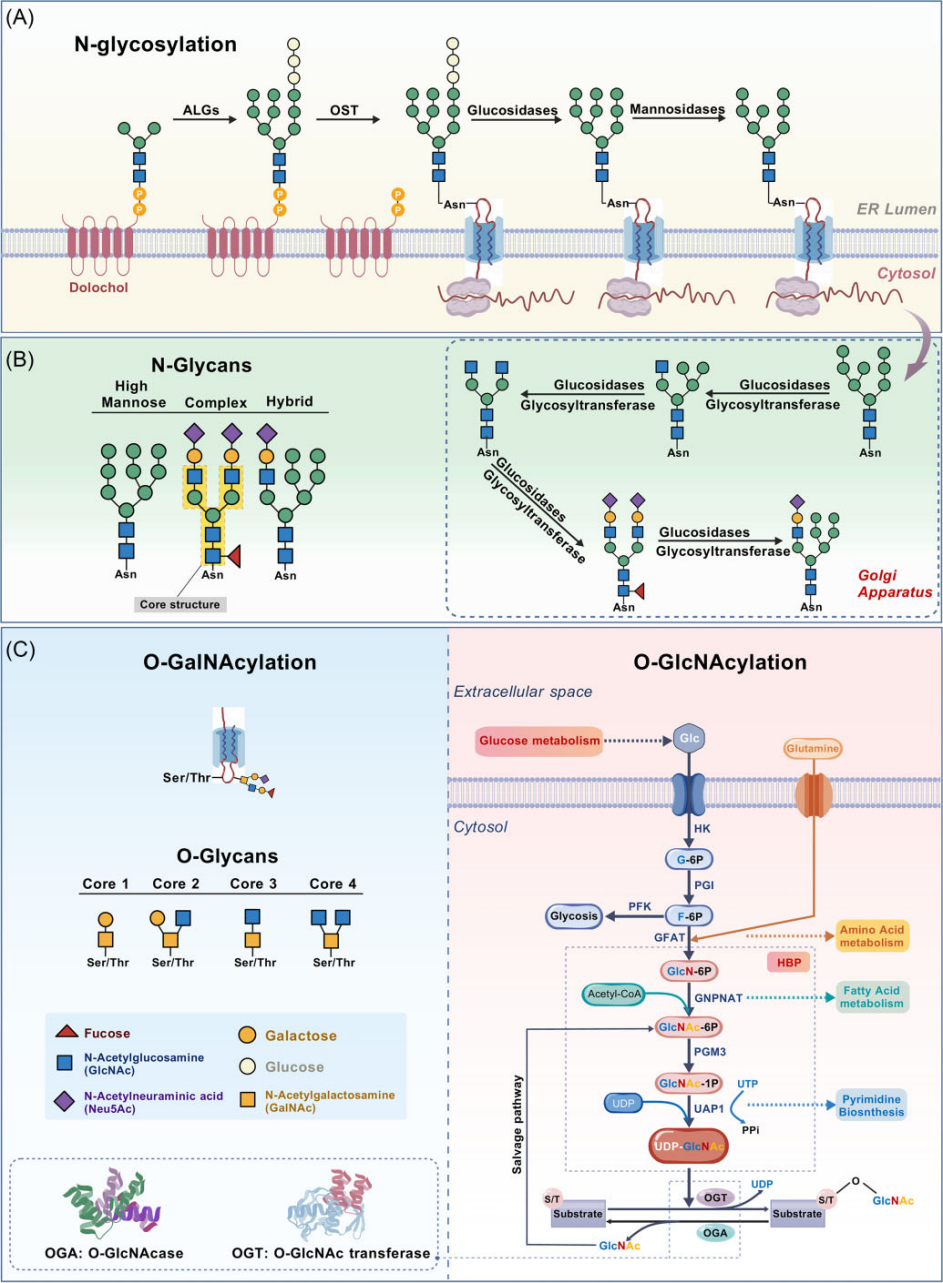
Schematic illustration of *N/O*-glycosylation processes and structures in cells. (**A**) Biosynthesis of *N*-glycans initiates in the ER. Glycosyltransferases catalyze the attachment of glycan chains to asparagine residues of proteins, resulting in the formation of glycoproteins. These glycoproteins are then transported through the membranes of Golgi apparatus, where they undergo further processing and modifications to acquire diverse glycan structures. (**B**) Major types of *N*-glycans on eukaryotic cell surfaces. High-mannose type *N*-glycans retain unprocessed mannose residues due to bypassing Golgi processing. Complex type *N*-glycans exhibit 2–5 branched antennae with terminal modifications (e.g. sialylation) initiated in the medial-Golgi and finalized in the trans-Golgi compartments. Hybrid type *N*-glycans feature one processed branch (modified in the Golgi) alongside unmodified mannose-rich branches. (**C**) Overview of the hexosamine biosynthetic pathway (HBP) and *O*-GlcNAcylation. HBP is a small branch of glycolysis where glucose (Glc) is converted into fructose-6-phosphate (F-6P) through the initial two steps that are shared with the glycolysis pathway. Only 2%–3% of F-6P enters HBP. Once Glc enters the cell, it is rapidly phosphorylated to glucose-6-phosphate (G-6P) by hexokinase (HK). Subsequently, G-6P is isomerized by phosphoglucose isomerase (PGI) to produce F-6P, which serves as a substrate for either phosphofructokinase (PFK) or glucosamino-fructose aminotransferase (GFAT) within the glycolytic pathway. This reaction represents a rate-limiting step in the HBP. GFAT utilizes glutamine as an amine donor to generate glucosamine 6-phosphate (GlcN-6P). It is then *N*-acetylated by glucosamine 6-phosphate *N*-acetyltransferase (GNPNAT, GNA1) to generate *N*-acetyl-glucosamine 6-phosphate (GlcNAc-6P). This crucial step requires acetyl-CoA as an acetyl donor. GlcNAc-6P is then converted into *N*-acetylglucosamine-1-phosphate (GlcNAc-1P) by acetylglucosamine phosphate mutase (PGM3). UDP-*N*-acetylgalactosamine (UDP-GalNAc) undergoes epimerase-mediated conversion to uridine diphosphate-*N*-acetylglucosamine (UDP-GlcNAc). Using UTP as a nucleotide donor, UDP-*N*-acetylglucosamine pyrophosphorylase (UAP1) generates UDP-GlcNAc. UDP-GlcNAc is the substrate for *O*-GlcNAc transferase (OGT) leading to the formation of O-linked β-*N*-acetylglucosamine (*O*-GlcNAc)-modified proteins. β-*N*-acetylglucosaminidase (OGA) catalyzes the removal of *O*-GlcNAc from the proteins. Free GlcNAc can be recovered by the GlcNAc salvage pathway. This pathway converts GlcNAc into GlcNAc-6P, which can be utilized by HBP. The figure was created with BioGDP (https://www.biogdp.com).

As one of the most evolutionarily conserved protein modifications in eukaryotes, *N*-glycosylation is characterized by the β-1,4-glycosidic linkage of *N*-acetylglucosamine (GlcNAc) to the side chains of asparagine (Asn). This intricate process is carried out through coordinated enzymatic effort (such as α-mannosidases and *N*-acetylglucosaminyltransferases) within the ER–Golgi system [8]. Initiated in the ER, *N*-glycan biosynthesis involves core glycan assembly followed by stepwise modifications in the Golgi apparatus [14, 19], ultimately yielding structurally diverse mature *N*-glycans [20] (Fig. 1A). Based on terminal sugar composition and branching patterns, *N*-glycans can be classified into three subtypes: (i) high-mannose (Man) type (retaining the core pentasaccharide Man3-GlcNAc2-Asn with Man5-9 branches); (ii) complex type [multi-antennary structures with GlcNAc, galactose (Gal), and sialic acid extensions); and (iii) hybrid type (combining features of both high-mannose and complex types) [21, 22] (Fig. 1B). This modification is critical for ensuring proper protein function by regulating folding efficiency, enhancing structural stability, and modulating antigenic activity within the ER–Golgi apparatus [23]. Emerging research has established a strong association between aberrant *N*-glycosylation and multiple pathological conditions, including cancers, cardiovascular diseases, metabolic disorders, and kidney diseases [12, 24–27]. In the cardiovascular system, *N*-glycans modulate L- and T-type Ca^2+^ channel function by altering conformational stability, thereby influencing calcium influx and cardiomyocyte excitation–contraction coupling [17]. In diabetes, defective *N*-glycosylation of pancreatic β-cell glucose-sensing receptors impairs insulin secretion by reducing responsiveness to hyperglycemic stimuli [28]. Renal studies have revealed that bisected *N*-glycans with core fucosylation correlate positively with DKD severity, whereas galactosylation exhibits an inverse relationship. Mechanistically, multi-branched *N*-glycans exacerbate podocyte injury by strengthening IgG–FcγRIIIa interactions and activating the complement alternative pathway [29].

### *O*-Glycosylation

*O*-Glycosylation refers to the covalent attachment of glycans via *O*-glycosidic bonds to the hydroxyl groups of serine (Ser), threonine (Thr), or less commonly tyrosine (Tyr) residues. Its biological functions are closely associated with specific modification subtypes [30]. Compared to *N*-glycosylation, *O*-glycosylation exhibits greater complexity and diversity, with the initial monosaccharides directly linked to proteins including GlcNAc, fucose (Fuc), mannose, glucose (Glc), Gal, and xylose (Xyl) [11]. Based on core monosaccharide differences, *O*-glycosylation is primarily classified into two categories: *O*-GalNAcylation and *O*-GlcNAcylation [31] (Fig. 1C). *O*-GalNAcylation (mucin-type *O*-glycans) is predominantly localized on cell membranes and in secreted glycoproteins. Its biosynthesis initiates with the attachment of GalNAc to Ser/Thr residues catalyzed by Golgi-localized GalNAc-transferases (GALNTs), forming the core GalNAc-O-Ser/Thr structure [32–34]. Subsequent elongation by β-1,3-galactosyltransferase (C1GALT1) forms core 1 (Galβ1–3GalNAc) or core 3 (GlcNAcβ1–3GalNAc) structures, which can further branch into core 2 (Glcβ1–6GalNAc) and core 4 (GlcNAcβ1–6GlcNAcβ1–3GalNAc) structures. These core structures serve as scaffolds for complex glycan extensions, mediating extracellular matrix (ECM) assembly and intercellular signaling. In contrast, *O*-GlcNAcylation (non-mucin *O*-glycans) is dynamically regulated by *O*-GlcNAc transferase (OGT) and *O*-GlcNAcase (OGA) [11, 35, 36]. Crucially, *O*-GlcNAcylation is tightly integrated with the hexosamine biosynthesis pathway (HBP), a metabolic hub that links glucose, amino acid, fatty acid, and nucleic acid metabolism. In HBP, fructose-6-phosphate is converted into glucosamine-6-phosphate via glutamine fructose-6-phosphate aminotransferase (GFAT), the rate-limiting enzyme, ultimately yielding uridine diphosphate *N*-acetylglucosamine (UDP-GlcNAc). OGT catalyzes the formation of β-*O*-glycosidic bonds between UDP-GlcNAc and Ser/Thr residues of target proteins [37], while OGA hydrolyzes these bonds to remove GlcNAc moieties (Fig. 1C). Predominantly located in nuclear, cytoplasmic, and mitochondrial compartments, *O*-GlcNAcylation regulates transcription, translation, protein–protein interactions, and subcellular trafficking to maintain cellular homeostasis [35, 36, 38]. Specifically, it modulates protein stability, subcellular localization, signal transduction efficiency, chromatin remodeling, and mitochondrial function to govern diverse biological processes [39, 40]. Consequently, *O*-GlcNAcylation is extensively involved in critical pathways such as immune recognition, apoptosis, metabolic homeostasis, and tumorigenesis [41–43]. For example, in colorectal cancer, elevated OGT expression in metastatic lymph nodes enhances EZH2 stability via *O*-GlcNAcylation, thereby promoting tumor cell invasion and migration [44]. In renal diseases, aberrant *O*-glycosylation contributes to multiple injury mechanisms. In IgAN, defective *O*-glycosylation promotes glomerular deposition of pathogenic IgA complexes, exacerbating renal damage [45, 46]. In DKD, hyperglycemia-induced *O*-glycosylation promotes basement membrane thickening, cellular hypertrophy, and podocyte dysfunction through the modification of specific proteins [47, 48]. Importantly, pharmacological inhibition of key enzymes in the *O*-glycosylation pathway has been shown to mitigate glucotoxicity and delay progression of end-stage renal disease (ESRD).

## Analytical methods for glycosylation

The high complexity of glycan structures poses severe challenges for the precise qualitative and quantitative analysis of glycoproteomics in kidney diseases. Extracting disease-specific glyco-signatures from complex biological samples urgently requires advanced technologies capable of both structural elucidation and quantitative profiling. Currently, released glycans from glycoproteins can be indirectly detected using lectin microarrays, or directly analyzed through separation techniques such as capillary gel electrophoresis (CGE), high-performance liquid chromatography (HPLC), and mass spectrometry (MS). These methodologies differ fundamentally in their operational principles and application domains, each offering unique advantages and limitations (Table 1). Additionally, the analysis of intact glycopeptides from glycoproteins is even more challenging. Despite technological advances, critical limitations persist: (i) low sensitivity for trace glycopeptides in clinical samples; (ii) inability to resolve glycan microheterogeneity and structural isomers; (iii) lack of standardized databases for automated glycopeptide annotation; and (iv) poor integration of glycoproteomics with spatial multi-omics in kidney tissues. These analytical constraints have been systematically evaluated in recent comprehensive reviews. Collectively, while current glycoproteomic platforms provide the technical foundation for elucidating disease mechanisms and discovering potential glyco-biomarkers, their clinical translation necessitates overcoming these four core limitations.

**Table 1.** Advantages and disadvantages of several technologies for glycan analysis.

Method	Principle	Applications	Advantages	Disadvantages
Lectin microarray	Specific binding of lectin–glycan	Qualitative screening of glycans, high-throughput differential analysis	High-throughput, simple operation, low cost	Low resolution, depends on known lectin recognition
CGE	Separation of glycans driven by electric field	Separation of low-molecular-weight glycans, isomers	High resolution, high sensitivity, fast speed, efficient separation	Low sample processing volume, poor separation reproducibility
LC^a^	Separation by chromatographic column	Separation of glycans, quantitative analysis, purity detection	Strong separation ability, high sensitivity, high resolution, high degree of automation, compatible with MS	Complex pretreatment, high cost
MS	Separation of glycans by mass-to-charge ratio	Precise qualitative and quantitative analysis of glycans, identification of glycosylation sites	High sensitivity, strong structural analysis ability, high-throughput	High cost, complex data interpretation, strict pretreatment

^a^Liquid chromatography.

### Lectin microarray

Lectins are a diverse family of glycan-binding proteins derived from plants, invertebrates, and vertebrates [49]. Their unique capacity to recognize specific carbohydrate motifs (e.g. α2,6-sialylation, core fucosylation) has established them as essential tools for decoding glycan structures in glycoproteomics. Leveraging this specificity, lectin microarray technology has emerged as a high-sensitivity platform that immobilizes diverse lectins on solid-phase substrates to profile glycans via fluorescence-based detection [50]. Unlike traditional analytical methods, lectin microarrays do not require glycan release or enzymatic digestion, making them suitable for rapid glycan profiling in complex biological samples. Its advantages include operational simplicity, high throughput, high sensitivity, compatibility with complex samples, and elimination of pre-separation steps for glycans. Different lectins can specifically recognize and bind to distinct glycan structures [51]. However, critical limitations hinder nephrology applications: lectins exhibit cross-reactivity for similar glycans, lack quantitation capability, and fail to distinguish structural isomers, which compromises specificity in heterogeneous renal samples. Additionally, the current repertoire of mammalian lectins is incomplete, failing to cover all human glycan structures. This hinders detection of novel or low-abundance glycans and necessitates the discovery of new lectin probes [52]. For renal biomarker studies, lectin arrays remain primarily a discovery tool; clinical adoption requires validation by orthogonal methods (e.g. MS) due to false-positive risks. Integrating lectin-based immunohistochemistry with MS imaging enables spatial mapping of glycan distributions with enhanced precision [53, 54]. Continued innovation in these technologies will drive breakthroughs in biomedical research.

### Capillary gel electrophoresis

Capillary gel electrophoresis (CGE) is a liquid-phase separation methodology that utilizes polymeric gel matrices as the stationary phase. This technique relies on electrophoretic migration through a 3D polymeric network where differential migration velocities arise from variations in glycan hydrodynamic volume, conformational states, and charge-to-mass ratios [55]. Through the sieving effect of the gel matrix, CGE enables efficient fractionation of glycan mixtures and precise discrimination of molecular weight and conformational variations among glycans [55]. To enhance detection sensitivity, glycans are typically fluorescently labeled prior to analysis. When coupled with laser-induced fluorescence detection, this platform achieves sensitive profiling of trace-level glycans (<1 pmol) with minimal sample consumption, making it ideal for quantitative characterization of intricate glycan populations [56]. For enhanced analytical power, CGE is often integrated with mass spectrometry or nuclear magnetic resonance to improve the sensitivity and structural resolution of glycomic analysis [57]. Nevertheless, CGE's reliance on specialized instrumentation and low throughput severely limits its utility in large-scale renal cohort studies or clinical settings. Currently, CGE has been successfully applied in clinical research, such as the characterization of *N*-glycans in IgG-Fc from tuberculosis patients, where it has played a critical role in biomarker discovery and pharmaceutical development [58]. In nephrology, its niche remains analytical validation rather than routine diagnostics.

### Liquid chromatography

Liquid chromatography (LC) remains a cornerstone technique for the separation and detection of glycans. Ultra-HPLC (UHPLC) systems, featuring sub-2 µm stationary-phase particles and narrow-bore columns under high-pressure conditions, markedly improve separation efficiency [59, 60]. Compared to conventional LC, UHPLC achieves >9-fold faster separation, 2-fold higher peak capacity, and 3–5-fold enhanced sensitivity [61, 62]. It is widely used in the analysis of free glycans, which requires the release of glycans from protein carriers before separation and detection, and corresponding sample pretreatment is necessary to obtain high-quality results. The hydrophilic interaction–ultra performance liquid chromatography (HILIC–UPLC) system has emerged as a powerful tool, enabling highly sensitive detection of *N*-glycans via fluorescence labeling strategies [60]. For example, Rudd *et al*. developed an automated ultrafiltration-based *N-*glycan analysis workflow that integrated UPLC with HILIC and fluorescence detection to enable parallel processing of 768 samples in a single run, emphasizing rapidity and simplicity throughout the workflow [63, 64]. Tharmalingam *et al*. further advanced the field by designing an automated UPLC-based sample preparation protocol with real-time monitoring capabilities, enabling dynamic tracking of glycosylation changes in recombinant proteins within cell cultures [65, 66]. Driven by its high resolution, rapid separation capability, sensitivity, and compatibility with MS, UHPLC has become central to glycoprotein glycosylation analysis [67]. The combination significantly enhances deep characterization of complex samples, facilitates high-throughput screening, and enables dynamic tracking in glycosylation studies [68]. However, UHPLC struggles to resolve sialic acid linkage isomers in renal glycoproteins, which is a critical gap for identifying disease-specific biomarkers [69].

### Mass spectrometry

**Figure 2.**
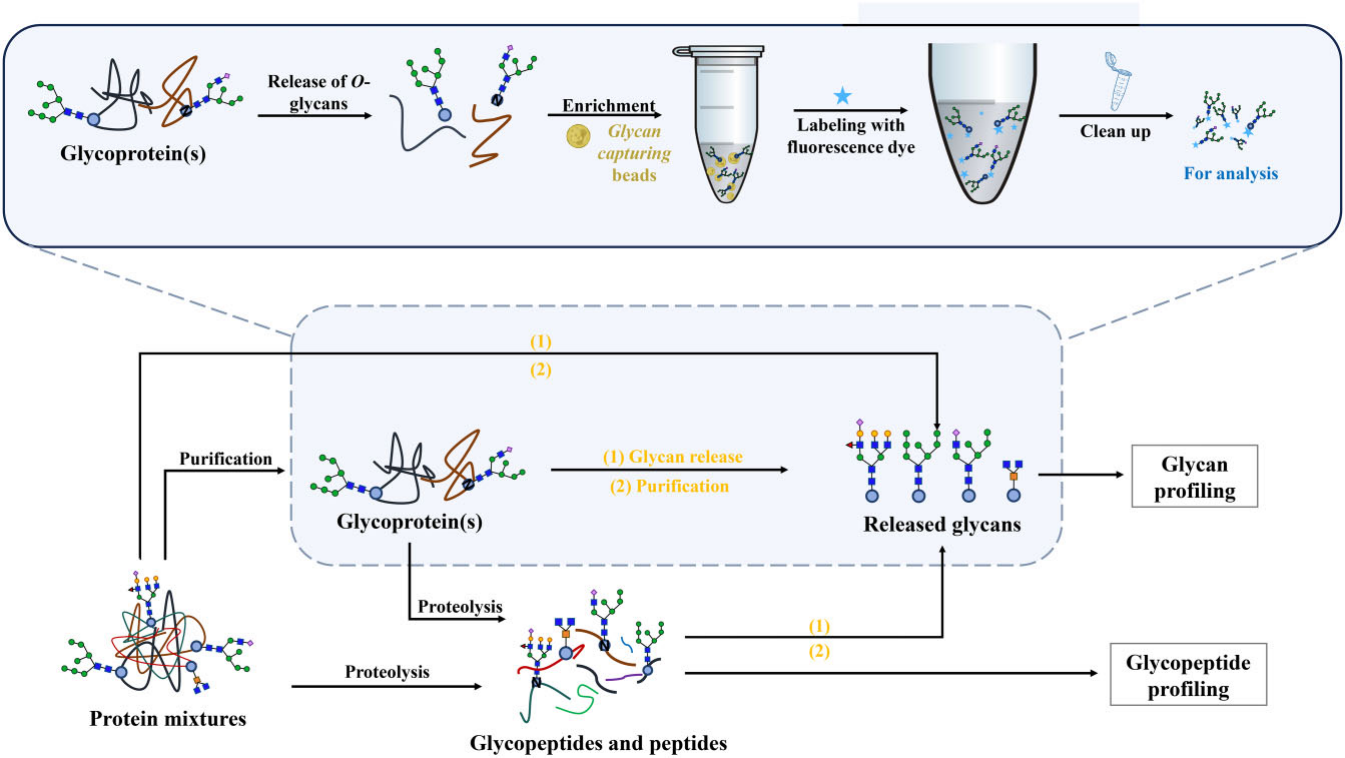
Workflow of glycosylation analysis. The pipeline includes sequential steps: sample collection, protein extraction, glycoprotein enrichment, reduction and alkylation, enzymatic digestion, glycopeptide enrichment, glycan release, data analysis, and visualization. Figure created with BioGDP (https://www.biogdp.com).

Mass spectrometry has emerged as a pivotal technology in glycomics, offering unparalleled advantages such as high sensitivity, resolution, rapid analysis, and compatibility with multiple separation techniques to simultaneously resolve glycan structures and quantify their abundance [70, 71]. When coupled with advanced separation techniques, MS enables comprehensive glycosylation profiling, driving breakthroughs in biomarker discovery and mechanistic research [59]. Among them, matrix-assisted laser desorption/ionization time-of-flight mass spectrometry (MALDI-TOF-MS) stands out for its operational simplicity and high sensitivity, making it ideal for high-throughput analysis of glycan molecular weights. However, it faces limitations in separating complex samples and is susceptible to matrix interference [72]. Coupled with UPLC, electrospray ionization mass spectrometry (ESI-MS) facilitates glycan separation, making it suitable for analyzing low-abundance glycans and providing structural fragmentation information. However, this technique demands high sample purity and involves complex interpretation of multi-charged ions [73, 74]. Ion mobility spectrometry–mass spectrometry (IMS–MS) enables isomers identification without derivatization or enzymatic reactions, offering additional insights into molecular conformation and collision cross-sectional area information to enhance glycan structure resolution. However, it is hindered by high instrument costs and complex data processing requirements [75]. Prior to MS analysis, samples typically undergo protein extraction, denaturation, enzymatic digestion, glycopeptide enrichment, glycan release, and fluorescent/isotope labeling. Finally, glycan structures are elucidated by tandem mass spectrometry (MS/MS), with data analysis and visualization supported by databases [76] (Fig. 2). Among these approaches, LC–MS/MS has become indispensable for glycoprotein research, enabling simultaneous acquisition of glycan structures, peptide sequences, and glycosylation-site information. Compared to traditional automated chemical detection systems, LC–MS/MS offers superior accuracy, speed, and comprehensiveness [77]. In biomedicine, LC–MS/MS is widely used for detecting clinical diagnostic biomarkers [78]. It can effectively resolve diverse *N*-glycan forms in complex mixtures while providing essential details on glycosites and glycoforms [79]. Nevertheless, broader clinical implementation, including in fields like nephrology, encounters persistent barriers. Key challenges include: high costs and technical expertise requirements, limiting routine use; a lack of standardized protocols for processing biofluid and tissue samples (e.g. urine, renal tissue); and incomplete spectral libraries hindering automated glycopeptide annotation. To realize the full clinical potential of LC–MS/MS for glycoprotein-based diagnostics and research, these obstacles must be tackled. Despite its power, LC–MS/MS faces significant barriers to clinical nephrology adoption: the high cost and technical expertise requirements preclude routine use; additionally, standardized protocols for processing renal samples like urine and tissue are lacking; and furthermore, incomplete spectral libraries hinder automated renal glycopeptide annotation. Encouragingly, continuous innovations relevant to glycoprotein analysis, such as advancements in MS fragmentation techniques, the development of automated sample-handing tools, and adoption of high-resolution mass spectrometers have progressively enhanced MS efficacy in glycomics. These developments hold promise for overcoming the current limitations in nephrology by enabling deeper structural characterization of renal glycopeptides or glycans, higher-throughput screening suitable for clinical settings, and more precise quantification, ultimately facilitating the establishment of standardized workflows and more comprehensive spectral libraries.

## Role of glycosylation in kidney diseases

Protein glycosylation is fundamental to renal physiology, governing the stability and activity of key proteins in the glomerular filtration barrier. Dysregulated glycosylation of these glycoproteins has been implicated in various nephropathies [36]. For example, the hallmark event in IgAN is the production of Gal-deficient IgA1 (Gd-IgA1). Aberrant *O*-glycosylation at the hinge region (HR) of IgA1 accumulates in the mesangial regions, triggering complement activation and subsequent renal injury [80]. DKD is characterized by hyperglycemia-induced *O*-GlcNAcylation dysregulation, which disrupts protein functionality to exacerbate ECM accumulation, podocyte effacement, and tubular atrophy. In RCC, the splicing factor DDX39B promotes expression of metastasis-associated genes via *N*-glycan biosynthesis pathways, facilitating tumor invasion and metastasis [81–83]. These findings collectively underscore glycosylation dysregulation as a central mechanistic driver in renal disease pathogenesis. In this section, we comprehensively discuss the multifaceted roles of glycosylation modifications across various renal disorders.

### Molecular mechanisms of aberrant *O*-glycosylation in driving IgAN pathogenesis and immune-mediated renal injury

**Figure 3.**
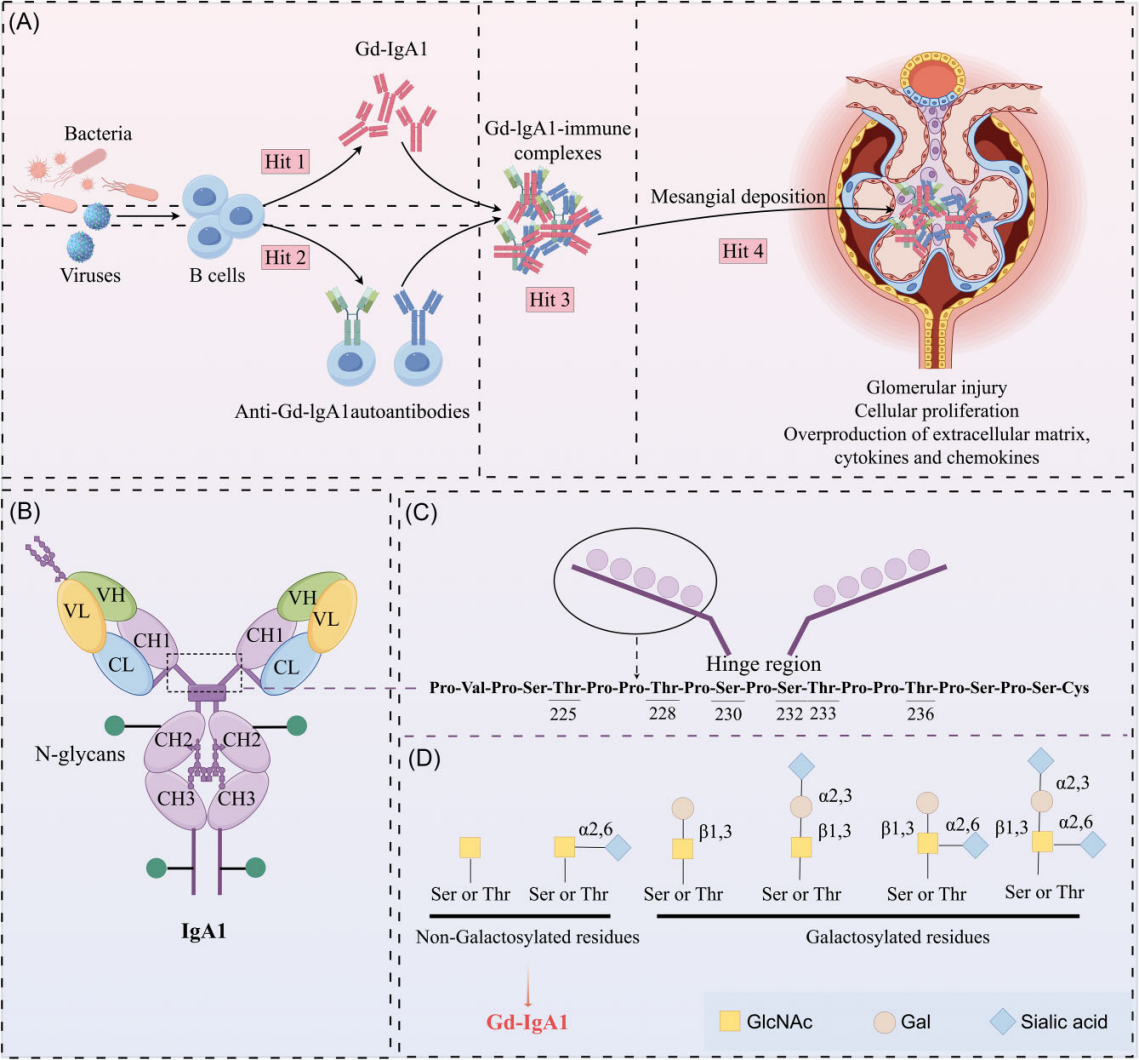
Mechanisms of abnormal glycosylation in IgAN. (**A**) The four-hit hypothesis of IgAN pathogenesis. Hit 1: increased Gd-IgA1 production. Hit 2: autoantibody formation. Hit 3: immune complexes formation. Hit 4: mesangial deposition. (**B**) *N*-Glycosylation in the IgA1 heavy chains. Each IgA1 heavy chain contains two *N*-glycans, one located in the CH2 domain and the other in the tail segment. (**C**) *O*-Glycosylation in the IgA1 HR. The HR of IgA1 harbors 9 Ser/Thr residues as potential *O*-glycosites. (**D**) Structures of *O*-glycan in IgA1. The figure was created with Figdraw (https://www.figdraw.com).

IgAN is the most common primary glomerulonephritis and a leading cause of ESRD [84]. Observational studies indicate that most patients are at risk of developing renal failure during their lifetime [85]. While its etiology remains incompletely elucidated, IgAN is fundamentally an immune-mediated disorder characterized by glomerular mesangial IgA deposition, governed by the “four-hit hypothesis” which directly links aberrant IgA1 *O*-glycosylation to downstream renal injury [84] (Fig. 3A). Crucially, the abnormal *O*-glycosylation of the IgA1 HR, particularly Gd-IgA1, represents the molecular hallmark that initiates this cascade reaction [86]. IgA is one of the most heavily glycosylated immunoglobulins, existing predominantly as IgA1 and IgA2 subtypes. Despite sharing high sequence homology, these subtypes differ markedly in HR length and glycosylation patterns (Fig. 3B). Human IgA1 features two *N*-glycosites situated in the CH2 region and tailpiece at asparagine residues Asn263 and Asn459, respectively. The IgA1 HR contains two octapeptide repeats with 3–6 core 1 *O*-glycans attached to Ser/Thr residues, whereas IgA2 lacks *O*-glycosylation-capable HRs, retaining only *N*-glycosylation and limited sialylation [87, 88] (Fig. 3C). The *O*-glycosylation of IgA1 occurs in the Golgi apparatus, where 9 Ser/Thr residues undergo stepwise enzymatic processing to form 3–6 *O*-glycans [89]. The *O*-glycosylation defect arises from disrupted enzymatic coordination in the Golgi apparatus. This process is initiated by the addition of GalNAc mediated by *N*-acetylgalactosaminyltransferase 2 (GalNAcT2) [90]. Subsequently, *O*-glycan chains can be extended through β-1,3 linkage of galactose to GalNAc mediated by C1GalT1, which requires the assistance of core 1,3-galactosyltransferase-specific molecular chaperone Cosmc [91, 92]. Reduced C1GalT1 activity, downregulated Cosmc expression, or increased GalNAc sialylation all lead to increased production of Gd-IgA1 [93, 94]. Sialic acid can be attached to Gal residues or directly modify GalNAc residues. If GalNAc is sialylated before galactosylation, the sialyl-Tn antigen (sTn) is formed, which hinders subsequent Gal addition and traps glycan chains in the sTn state, ultimately forming Gd-IgA1 [95, 96] Pathologically, reduced C1GalT1/Cosmc activity synergizes with premature sialylation of GalNAc residues (forming sTn), trapping glycans in incomplete states and directly generating Gd-IgA1 (Fig. 3D). This glycosylation aberration triggers a self-amplifying immune cascade. Gd-IgA1 is recognized by autoantibodies IgG and IgA, forming immune complexes that deposit in the kidneys. These complexes then activate two interlinked effector pathways. By activating the inflammatory response and complement system, this process induces renal inflammation, glomerular damage, and fibrosis, leading to the development of IgAN [84]. Clinically, serum Gd-IgA1 levels exhibit a strong correlation with histological severity and clinical progression, cementing its dual role as diagnostic biomarker and therapeutic target in IgAN [97–99].

### *O*-GlcNAcylation as a key pathomechanistic driver in DKD

DKD, the most prevalent microvascular complication of diabetes, represents a leading cause of morbidity and mortality in diabetic populations. Approximately one-third of individuals with type 1 diabetes and half of those with type 2 diabetes develop DKD, which has emerged as a major contributor to the rising global burden of CKD, accounting for nearly 50% of CKD cases and representing the primary etiology of ESRD [100–102]. In recent years, studies have confirmed that the HBP and *O*-GlcNAcylation play critical roles in DKD. Hyperglycemia increases flux through HBP by activating the expression and activity of GFAT, leading to increased production of UDP-GlcNAc and enhanced protein *O*-GlcNAcylation in renal cells [103, 104]. *O*-GlcNAcylation of proteins regulates the functions of renal cells including mesangial cells, podocytes, and proximal tubular cells, ultimately triggering a series of pathological responses such as renal cell injury and renal interstitial fibrosis [105]. This section synthesizes current insights into how glycosylation dysregulation contributes to DKD initiation and progression.

**Figure 4.**
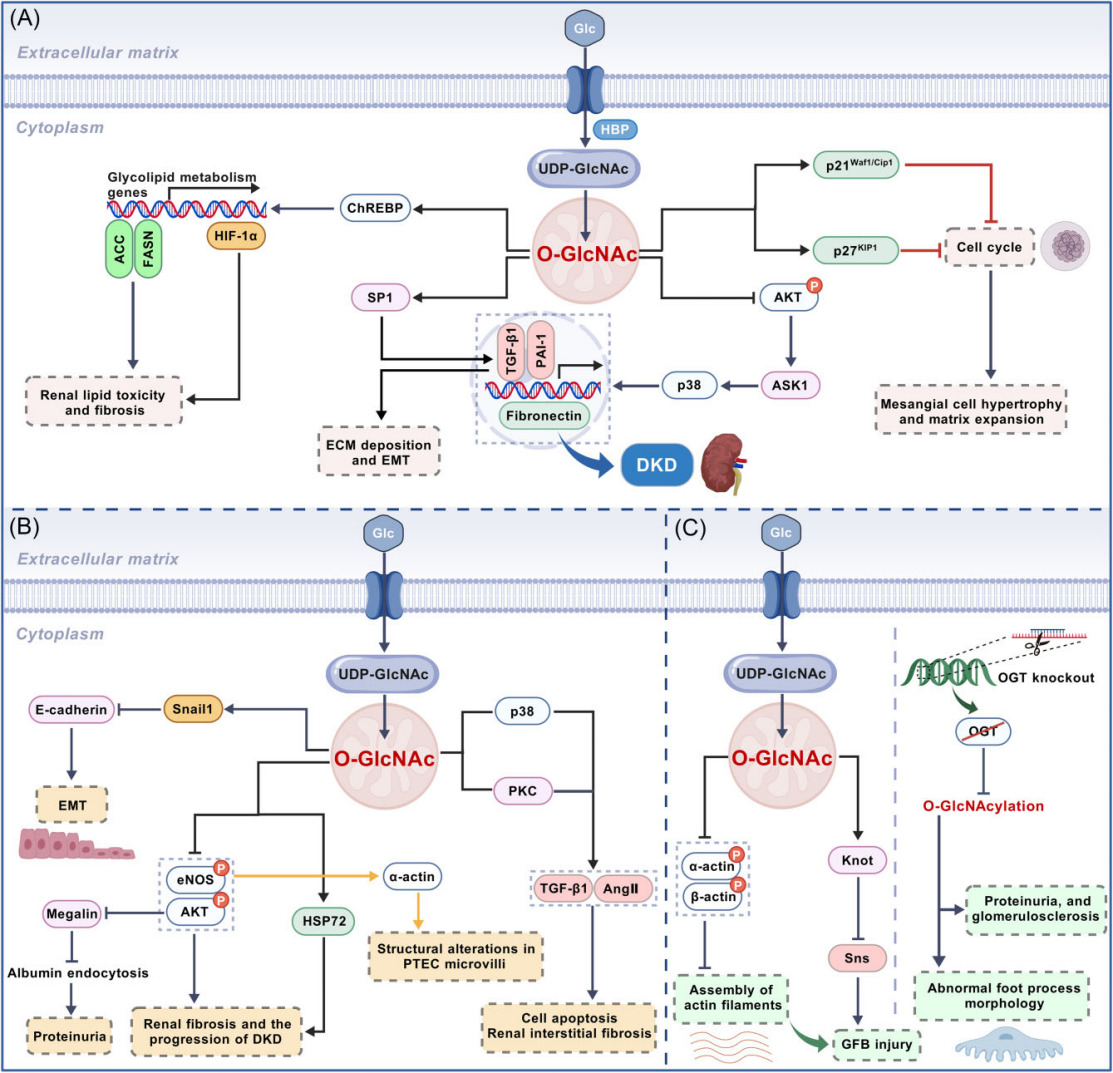
Mechanisms of abnormal *O*-GlcNAcylation in DKD. (**A**) In mesangial cells, high glucose promotes renal fibrosis through multiple mechanisms: regulating ChREBP via *O*-GlcNAcylation modification leading to renal fibrosis and lipotoxicity; inducing mesangial cell hypertrophy and matrix proliferation through the regulation of p21Waf1/Cip1 and p27KIP1; inhibiting AKT phosphorylation and promoting SP1 expression, thereby driving the release of downstream factors such as PAI-1, fibronectin, and TGF-β1, which ultimately mediates the process of renal fibrosis. (**B**) In renal tubular cells, high glucose promotes renal fibrosis through multiple mechanisms including regulating Snail protein via *O*-GlcNAcylation modification to induce EMT; triggering tubular interstitial fibrosis by inducing the expression of p38 and PKC; and inhibiting the phosphorylation of eNOS and AKT, upregulating the expression of HSP72, and exacerbating the process of renal fibrosis. (**C**) In podocytes, high glucose inhibits the phosphorylation levels of α-actin and β-actin through *O*-GlcNAcylation modification, while activating transcription factor Knot and downregulating the expression of nephropathy protein homolog Sns, ultimately leading to damage of the podocyte filtration barrier. Additionally, OGT-knockout mice gradually exhibit pathological changes such as podocyte foot process morphology abnormalities, proteinuria, and glomerulosclerosis. “↑” Indicates activation, stimulation, or promotion, whereas “⊥” indicates inhibition, suppression, or decrease. Abbreviation: HIF-1α, Hypoxia-inducible factor 1-α; ACC, acetyl-CoA carboxylase; FASN, fatty acid synthase; SP1, specificity protein 1; p21^Waf1/Cip1^, cyclin-dependent kinase inhibitor 1A; p27^Kip1^, cyclin-dependent kinase inhibitor 1B; AKT, protein kinase B; ASK1, apoptosis signal-regulating kinase 1; Snail1, snail family transcriptional repressor 1; eNOS, endothelial nitric oxide synthase; HSP72, heat shock protein 72; p38, p38 mitogen-activated protein kinase; PKC, protein kinase C; Ang II, angiotensin II; GFB, glomerular filtration barrier. The figure was created with BioGDP (https://www.biogdp.com).

Mesangial cells, essential components of the glomerulus, maintain glomerular structural stability and physiological functions through multiple mechanisms. Notably, abnormal *O*-glycosylation within mesangial cells drives the pathogenesis of DKD by promoting ECM deposition, mesangial matrix hyperplasia, and renal fibrosis. Carbohydrate response element binding protein (ChREBP), a key transcriptional regulator of glucose–lipid metabolism, regulates the transcription of genes related to glycolipid metabolism by recognizing carbohydrate response elements (ChoRE) in the promoter regions of target genes [106]. In mesangial cells, high glucose enhances ChREBP *O*-GlcNAcylation and upregulates renal acetyl-CoA carboxylase (ACC) and fatty acid synthase (FASN) to exacerbate lipid toxicity, prompting HIF-1α to further intensify renal fibrosis [107, 108] (Fig. 4A). Studies have shown that the significantly elevated *O*-GlcNAcylation in glomerular mesangial cells can inhibit protein kinase B (AKT) phosphorylation, activate the upstream ASK1 kinase, and promote the phosphorylation of p38 mitogen-activated protein kinase (MAPK), ultimately driving the expression of downstream plasminogen activator inhibitor-l (PAI-1), fibronectin, and transforming growth factor-β1 (TGF-β1), contributing to excessive matrix accumulation in DKD [109, 110]. Critically, these pathways exhibit functional synergy. Among them, TGF-β1 not only directly exacerbates renal interstitial fibrosis by promoting ECM deposition and inducing epithelial–mesenchymal transition (EMT) [111], but also activates p38 MAPK signaling, thereby amplifying profibrotic and inflammatory responses. Meanwhile, PAI-1 impedes ECM degradation by inhibiting plasminogen conversion, establishing a coordinated matrix accumulation network [112] (Fig. 4A). Additionally, the specific transcription factor SP1 regulates the expression of PAI-1 and TGF-β1 by binding to specific regions of their promoters. *O*-GlcNAcylation of SP1 directly enhances both genes’ transcription, further promoting the development of DKD [113] (Fig. 4A). Masson *et al*.'s *in vitro* studies revealed that HBP activation by glucosamine (GlcN) arrests mesangial cells at the G0/G1 phase via upregulation of cyclin-dependent kinase inhibitor p21^Waf1/Cip1^ and p27^KIP1^, leading to mesangial cell hypertrophy and matrix expansion [114, 115] (Fig. 4A). During the cell cycle process, the levels of OGT, OGA, and *O*-GlcNAc undergo significant fluctuations. Therefore, *O*-GlcNAc glycosylation modifications and alterations in HBP flux are increasingly becoming crucial factors in regulating cell cycle progression. Additionally, increased *O*-GlcNAcylation of cellular proteins inhibits the endogenous and intracellular cgalcium influx-induced swelling-dependent chloride channels currents, preventing the regulatory volume decrease following cell swelling, which contributes to the development of DKD [116]. Inflammatory responses in DKD are closely linked to high glucose-induced *O*-GlcNAcylation. Elevated HBP flux in mesangial cells enhances NF-κB transcriptional activity via *O*-GlcNAc modification, directly upregulating adhesion molecule 1 and amplifying inflammatory signaling through increased NF-κB–DNA binding affinity, sustaining cytokine release and promoting glomerulosclerosis and fibrosis [117, 118].

Tubulointerstitial fibrosis (TIF), a hallmark of CKD progression to ESRD, involves EMT. Studies have found that *O*-GlcNAcylation contributes to EMT in TIF. As a potential substrate for *O*-GlcNAcylation, glucosamine can induce upregulation of RAF1 and promote EMT and migratory capacity of human proximal tubular human kidney 2 cells [119]. High glucose activates OGT to catalyze *O*-GlcNAcylation of snail family transcriptional repressor 1 (Snail1) protein in renal tubular epithelial cells. Snail1 induces EMT by inhibiting E-cadherin promoter activity and transcription, a process where high glucose reduces Snail1 phosphorylation while enhancing its *O*-GlcNAcylation [120] (Fig. 4B). Importantly, *O*-GlcNAcylation in tubular cells induces p38 MAPK and PKC expression, promoting TGF-β1 and angiotensin II production to induce cell apoptosis and renal interstitial fibrosis (RIF) [121] (Fig. 4B). Gellai *et al*. further demonstrated that hyperglycemia-induced *O*-GlcNAcylation exacerbates renal fibrosis and promotes DKD progression by inhibiting phosphorylation of endothelial nitric oxide synthase (eNOS) and AKT, while upregulating heat shock protein 72 (HSP72) [122]. Furthermore, *O*-GlcNAcylation-mediated suppression of eNOS and AKT phosphorylation also upregulates α-actinin and disrupts tubular microvilli structure, contributing further to renal damage in DKD [48, 123] (Fig. 4B). Megalin, an endocytic receptor in proximal tubule, impairs albumin reabsorption when dysfunctional, leading to proteinuria [124]. Silva-Aguiar *et al*. showed that high glucose elevates intracellular *O*-GlcNAcylation, which inhibits AKT phosphorylation. This ultimately decreases megalin expression, impairs albumin endocytosis in renal tubular cells, and triggers proteinuria [125–127]. Additionally, *O*-GlcNAcylation can disrupt retinoic acid homeostasis by reducing retinol signaling in cells cultured under normal glucose conditions, thereby promoting renal fibrosis [128]. Interestingly, although increased protein *O*-GlcNAcylation is generally considered nephrotoxic, some studies suggest it may also exert renal protective effects. For example, *O*-GlcNAcylation participates in renal tubular pathological processes by regulating lipid metabolic homeostasis. Sugahara *et al*. found that high glucose environments enhance *O*-GlcNAcylation in renal tubular epithelial cells of fasting or diabetic mice, promoting lipid catabolism to maintain renal energy homeostasis and function. In contrast, OGT knockout mice exhibit severe renal tubular damage due to impaired *O*-GlcNAcylation [129]. These findings indicate that high glucose-induced *O*-GlcNAcylation must be maintained within a moderate range to exert its effects, though this concept requires further validation.

During DKD pathogenesis, high glucose-induced *O*-GlcNAcylation drives podocyte injury, leading to morphological changes, cell detachment, and apoptosis of podocytes, which is a major cause of glomerular filtration barrier dysfunction and proteinuria [130]. OGT-mediated glycosylation is crucial for foot process maturation and stability [131]. Podocyte-specific OGT knockout causes abnormal foot process morphology, proteinuria, and glomerulosclerosis, confirming its structural necessity [131] (Fig. 4C). OGT-driven *O*-GlcNAcylation exacerbates podocyte injury in DKD through post-translational modification of key regulators in the ECM signaling pathway [132]. However, under high glucose, OGT-driven *O*-GlcNAcylation damages the glomerular filtration barrier. On the one hand, the elevated *O*-GlcNAcylation reduces the phosphorylation of cytoskeletal proteins such as α-actin and β-actin, hinders the assembly of actin filaments, and leads to pathological changes in the microvilli of the foot process and renal tubules [133]. On the other hand, this modification downregulates nephrin ortholog Sns by activating factor Knot transcription, further exacerbating podocyte filtration barrier damage [134] (Fig. 4C). Thus, *O*-GlcNAcylation exhibits a dual effect on podocytes, being essential for structural integrity but pathogenic when dysregulated, further emphasizing the need for its precise regulation in the prevention and treatment of DKD.

### IgA1 glycosylation abnormalities in Henoch-Schönlein purpura nephritis

**Figure 5.**
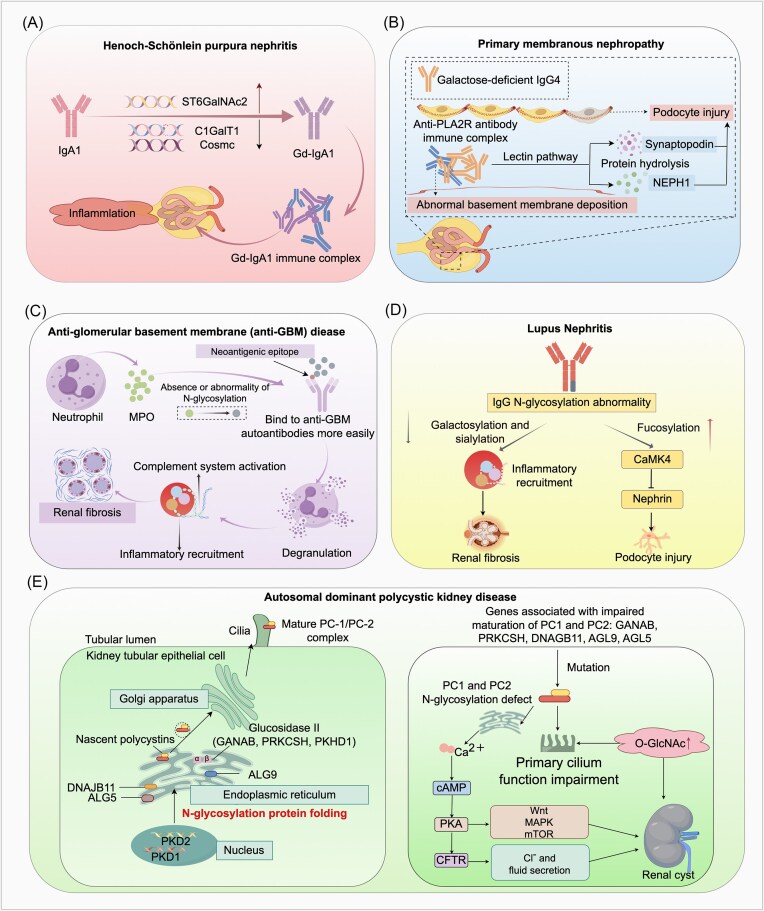
Schematic diagram of glycosylation in nephropathy pathogenesis. (**A**) Mechanism of Gd-IgA1 in HSPN. (**B**) Mechanism of abnormal IgG4 glycosylation in primary membranous nephropathy. (**C**) Mechanism of abnormal *N*-glycosylation of MPO in anti-GBM disease. (**D**) Mechanism of abnormal glycosylation in LN. (**E**) Mechanism of abnormal glycosylation in ADPKD. “↑” Indicates activation, stimulation, or promotion, whereas “⊥” indicates inhibition, suppression, or decrease. Anti-GBM, Anti-glomerular basement membrane; LN, lupus nephritis; NEPH1, nephrin-related protein 1; MPO, myeloperoxidase; CAMK4, calcium/calmodulin-dependent protein kinase IV; PC1, polycystin-1; PC2, polycystin-2; cAMP, cyclic adenosine monophosphate; PKA, protein kinase A; CFTR, cystic fibrosis transmembrane conductance regulator. The figure was created with Figdraw (https://www.figdraw.com).

Henoch-Schönlein purpura nephritis (HSPN) is the primary clinical manifestation of renal involvement in Henoch-Schönlein purpura (HSP), also known as IgA vasculitis [135]. It is an immune complex-mediated glomerulonephritis. Similar to IgAN, aberrantly glycosylated IgA1 plays a central role in pathogenesis [136]. The formation of these abnormal IgA1 molecules primarily results from dysregulated expression and loss of activity of glycosyltransferase. In HSPN, the expression of C1GalT1 and Cosmc is significantly downregulated, leading to a decreased galactosylation capacity of IgA1. Concurrently, a marked increase in ST6GalNAc2 expression elevates the sialylation level of IgA1. This premature sialylation impedes subsequent Gal addition, resulting in the formation of Gd-IgA1. Due to the absence of Gal in its O-glycan chains, Gd-IgA1 exposes novel antigenic epitopes. These epitopes are recognized by autoantibodies, forming circulating immune complexes (IgA1-ICs) [137]. These pathogenic Gd-IgA1 ICs deposit in the glomerular mesangium, activating the complement system and inflammatory responses, which ultimately lead to glomerular injury and interstitial fibrosis [137] (Fig. 5A). Elevated serum levels of Gd-IgA1 and its glomerular deposition are unique to HSPN and IgAN, distinguishing them from other IgA-deposition nephropathies [138]. This study demonstrated mesangial overexpression of the IgA1 receptor CD71 in both diseases, which binds polymeric IgA1 to promote mesangial deposition and activates the phosphatidylinositol 3-kinase (PI3K)-AKT-mammalian target of rapamycin pathway, driving mesangial proliferation. Furthermore, impaired binding of Gd-IgA1 to its receptor in patients delays the catabolism of IgA1 ICs, contributing to significantly elevated serum Gd-IgA1 levels [137, 139]. Critically, subendothelial IgA deposits in the kidneys of HSPN patients include not only Gd-IgA1 but also anti-endothelial cell antibody–IgA1 complexes, which are rarely observed in IgAN [140]. Additionally, serum and renal IgA1-ICs in HSPN patients exhibit higher molecular weights compared to those in IgAN patients [135, 141]. These distinct immunological and biochemical features provide important evidence for differentiating HSPN from IgAN.

### Pathogenic mechanism of IgG4 glycosylation defects in primary membranous nephropathy

Primary membranous nephropathy (pMN), a common pathological type of adult nephrotic syndrome (NS), is widely recognized as a classic autoimmune disease [142]. Its hallmark pathological features include abnormal deposition of immune complexes in the glomeruli. The immune system mounts an autoimmune response against podocyte surface antigens (such as the M-type phospholipase A2 receptor, PLA2R), generating specific antigen–antibody complexes that ultimately accumulate in the subepithelial space of the glomerular capillary walls beneath podocytes. Immunofluorescence staining reveals granular IgG deposits in the subepithelial region accompanied by effacement of podocyte foot processes [143]. Recent studies have indicated that abnormal glycosylation modifications play an important role in the pathogenesis of pMN. Elevated levels of Gal-deficient IgG4 in pMN patients correlate with anti-PLA2R antibody titers and podocyte injury [144]. Mechanistically, this Gal-deficient IgG4 can induce proteolysis of synaptopodin and nephrin-related protein 1 (NEPH1) in podocytes and disrupt cytoskeletal stability by activating the mannose-binding lectin pathway (MBL) [144] (Fig. 5B). In contrast, Fc galactosylation of IgG primarily influences classical complement pathway activation but does not affect MBL-mediated pathways *in vitro* [145]. Clizia *et al*. further systematically analyzed *N*-glycan profiles of IgG1, IgG2, and IgG4 in pMN patients, NS patients, and healthy controls [146]. While all IgG subtypes showed glycosylation alterations in pMN, only IgG4 exhibited disease-specific changes characterized by reduced galactosylation and hybrid glycans alongside increased fucosylation. This aberrant IgG4 glycosylation pattern was most pronounced in anti-PLA2R antibody-positive pMN cases, suggesting its potential as a biomarker for disease activity.

### Glycosylation abnormalities in anti-glomerular basement membrane

Anti-glomerular basement membrane (anti-GBM) disease is a severe autoimmune disorder characterized by elevated anti-GBM antibody titers, rapidly progressive glomerulonephritis, and pulmonary hemorrhage. Glycosylation aberrations represent critical molecular determinants in its pathogenesis. Autoantibodies targeting linear peptides of myeloperoxidase (MPO) are detectable in most anti-GBM disease patients, with >60% recognizing linear peptides on the MPO heavy chain, several of which correlate with disease severity [147]. Yu *et al*. first demonstrated that aberrant or absent *N*-glycosylation of MPO alters its antigenicity, exposing novel immunogenic epitopes [148]. Specifically, abnormally glycosylated MPO, including non-glycosylated forms or those with only *N*-GlcNAcylation, is more readily recognized by patient antibodies that predominantly target specific glycosites (N323, N355, and N391) [148]. Functionally, these pathogenic antibodies bind to MPO on neutrophil surfaces, inducing neutrophil degranulation and release of inflammatory mediators, ultimately causing endothelial damage, tissue inflammation, and driving renal fibrosis [148] (Fig. 5C). Complementing this, Reiding *et al*. discovered the heterogeneity of *N*-glycosylation in MPO and the potential for glycan epitopes [149]. They revealed an exceptional diversity of free oligosaccharides and occupancy levels across five glycosites and truncated high-mannose species in extracellular space. While the physiological significance of variable region glycosylation in autoantibodies remains poorly understood, studies show that although total IgG variable-region glycosylation does not differ significantly between active-phase anti-GBM patients and healthy individuals, affinity-purified anti-MPO antibodies exhibit higher variable-region glycosylation than total IgG, whereas anti-GBM antibodies display lower glycosylation, potentially modulating antigen-binding capacity [150]. Thus, neoepitopes exposed by aberrantly glycosylated MPO may contribute to renal injury mechanisms and represent promising therapeutic targets.

### Glycosylation dysregulation in lupus nephritis

Systemic lupus erythematosus (SLE) is a complex autoimmune disorder affecting multiple organ systems, characterized by IgG autoantibodies against nuclear antigens. These antibodies deposit in the kidneys, triggering lupus nephritis (LN)—the most severe complication of SLE and a major risk driver of morbidity and mortality [151, 152]. LN pathogenesis involves immune complex deposition, complement activation, autoantibody production, aberrant apoptosis, inflammatory cell infiltration, and genetic factors [153]. Emerging evidence highlights the critical role of abnormal protein glycosylation in LN development. As core immune effector molecules, IgG glycosylation patterns directly regulate LN progression. Reduced sialylation and galactosylation of IgG *N*-glycans may accelerate renal fibrosis progression by upregulating pro-inflammatory responses [154]. Moreover, fucosylation of IgG *N*-glycans upregulates calcium/calmodulin-dependent kinase IV (CaMK4) in podocytes, suppressing nephrin transcription and impairing podocyte integrity, whereas galactosylation inhibits CaMK4 to preserve nephrin expression and alleviate LN [155, 156] (Fig. 5D). The OGT gene, located at Xq13.1 near the X-inactivation center, has been implicated in SLE pathogenesis via overexpression [157]. OGT dynamically regulates immune cell function through *O*-GlcNAcylation for example, promoting the differentiation of CD4 + T cells into pro-inflammatory T helper cell 17 (Th17) subsets and enhancing interleukin-17 secretion, thereby exacerbating renal inflammation [158]. Sundararaj *et al*. demonstrated that neuraminidase exacerbates glomerular inflammation by reducing sialylated *N*-glycans and activating toll-like receptor 4 (TLR4)-p38/ERK MAPK signaling [159]. Integrating these findings, key regulatory glycosylation could be identified as a critical regulator of LN pathogenesis and potential target for therapeutic intervention.

### Glycosylation abnormalities in antineutrophil cytoplasmic antibody-associated vasculitis

Antineutrophil cytoplasmic antibody (ANCA)-associated vasculitis (AAV) is an autoimmune disorder characterized by small-vessel inflammation, with the kidneys being one of the most frequently affected organs. Over 75% of patients with AAV experience renal injury, manifesting as rapidly progressive glomerulonephritis [160]. This disease is defined by persistent serum ANCA, which is classified into two subtypes based on the target antigens: proteinase 3 (PR3-AAV) and MPO-AAV, corresponding to anti-PR3 and anti-MPO ANCA, respectively. PR3 and MPO are components of neutrophil granules and monocyte lysosomes. Mechanistically, after ANCA binds to these antigens, it can activate Fcγ receptors on neutrophils and monocytes, thereby triggering endothelial injury [161]. ANCA primarily belongs to the IgG subtype. Multiple studies have identified altered total IgG glycosylation in patients with anti-PR3 or anti-MPO ANCA. Total IgG sialylation is significantly reduced during active disease versus remission, with ANCA sialylation levels inversely correlating with disease activity [162–164]. Notably, Wojcik *et al*. observed a higher IgG Fc-dichotomy (ΔFc) in patients experiencing PR3-AAV relapse, with reduced Fc glycosylation preceding relapse, suggesting that lower IgG Fc-fucosylation may predict impending recurrence [165]. Experimentally, defucosylated anti-MPO ANCA glomerulonephritis attenuates renal injury in models, confirming the role of Fc glycosylation in the progression of AAV. Collectively, these findings reveal IgG glycosylation abnormalities as key determinants of AAV progression, offering novel insights into disease mechanisms and monitoring strategies.

### Glycosylation abnormalities drive pathogenesis of ADPKD via polycystin dysfunction

ADPKD, one of the most common inherited kidney diseases, is a leading genetic cause of renal failure [166]. It arises from mutations in *PKD1* or *PKD2* genes encoding polycystin-1 (PC1) and polycystin-2 (PC2), respectively [167, 168]. These mutations disrupt glycosylation of PC1 and PC2, impairing their trafficking and maturation to drive cyst formation [168].

For PC1 (a highly glycosylated protein with 61 potential *N*-glycosites in its extracellular domain), *PKD1* mutations hinder the recognition of PC1 by glycosyltransferases, leading to loss of glycosites or abnormal glycan processing. This disrupts PC1 localization to the cell membrane or its binding to PC2 [169, 170]. PC2, a cation channel protein mainly located to the ER, transduces signals by altering membrane potential or intracellular Ca^2+^ concentration. Its top domain contains five glycosites [171]. PC2 functions independently or binds to PC1 via its C-terminal coiled-coil domain to form a polycystin complex, mediating mechanical signal transduction and maintaining intracellular calcium homeostasis [172]. Abnormal PC2 glycosylation causes protein retention or degradation in the ER, preventing its trafficking to the cell membrane and impairing calcium channel function, thus disrupting calcium homeostasis [173]. PC1 and PC2 inhibit cyst formation in a dose-dependent manner; cysts develop when the concentration of PC1 or PC2 falls below a certain threshold [174, 175]. Loss or impairment of PC1/2 reduces Ca^2+^ influx and promotes cAMP accumulation, activating PKA to drive cell proliferation and stimulates cystic fibrosis transmembrane conductance regulator (CFTR)-mediated Cl^−^ secretion, thereby facilitating cyst expansion [176] (Fig. 5E). Additionally, decreased transient receptor potential vanilloid 4 (TRPV4) glycosylation inhibits channel activity, causing Ca^2+^ dysregulation to promote ADPKD [177]. PC1/2 localize to renal epithelial cells and the ER [178]. The ER biosynthesis pathway is crucial for the folding, quality control, and Golgi export of nascent polycystin precursors [179]. This process requires the binding of PC1 to PC2 and cleavage at the G protein-coupled receptor proteolytic site [179, 180]. *N*-Glycosylation within the ER relies on proteins encoded by genes such as *GANAB, DNAJB11, PRKSCH, ALG5*, and *ALG9*. Defects in these genes involved in the process disrupt *N*-glycosylation of polycystins, impair PC1 maturation, trigger the accumulation of misfolded proteins and ER stress, and drive cystogenesis [181] (Fig. 5E). For example, *GANAB* encodes the α subunit of glucosidase II, and its mutation causes defects in the initiation stage of *N*-glycosylation, leading to ADPKD [182]. *DNAJB11* encodes ERdj3, a glycoprotein in the ER lumen that acts as a chaperone for binding immunoglobulin protein (BiP). Its mutation disrupts BiP-mediated protein folding, causing PC1 to stagnate in the ER and leading to renal cysts and fibrosis [183]. Additionally, *PRKSCH* encodes the β subunit of glucosidase II, and its mutation may reduce the abundance of PC2 by affecting protein folding and/or translation efficiency [173]. *ALG5* encodes an ER-resident enzyme that catalyzes the transfer of glucose from UDP-glucose to dolichyl phosphate (Dol-P) to generate Dol-P-Glc, ensuring the integrity of the oligosaccharide chain precursor. The activity of *ALG5* is essential for maintaining normal *N*-glycosylation modifications. *ALG5* single allelic mutations can lead to insufficient Dol-P-Glc synthesis, causing *N*-glycosylation precursor defects and affecting PC1 maturation and transportation [184]. In patients with *ALG5* mutations, the levels of urinary uromodulin (Umod) in plasma and urine are reduced [184]. Due to insufficient glycosylation, Umod is retained in the ER, ultimately leading to TIF and renal cyst formation [185]. In addition to *ALG5, ALG9* encodes α-1,2-mannosyltransferase, which adds specific mannose molecules to the *N*-glycan precursors assembled in the ER lumen. Studies have reported that the deletion of the *ALG9* gene leads to *N*-glycosylation defects of PC1, causing impaired PC1 maturation and renal cysts [186].

Beyond ER defects, impaired primary cilia function constitutes another key pathogenic mechanism in ADPKD. These antenna-like sensory organelles critically regulate renal tubular homeostasis, wherein the PC1/2 ion-channel complexes mediate mechanosensory signals whose dysfunction drives cystogenesis [187, 188]. Elevated *O*-GlcNAcylation affects ADPKD pathology by dysregulating cilia formation and cell metabolism [189]. In juvenile and adult ADPKD mouse models, renal cysts, lengthening of renal cilia, inflammatory responses, and elevated *O*-GlcNAcylation levels were observed. Additionally, PC1/2 loss-of-function dysregulates Wnt, mammalian target of rapamycin, and MAPK pathways [190] (Fig. 5E). Supporting glycosylation's centrality, abnormal α3 integrin *N*-glycosylation in Pkd1^+/+^ mouse renal cells correlates with unique disialylglycan structures and cyst formation [191]. These studies collectively reveal a complex network linking glycosylation, signaling pathways, and ciliary functions, providing multi-dimensional insights into ADPKD pathologenesis.

### Multidimensional regulatory roles of glycosylation in RCC

RCC accounts for ∼2%–3% of adult malignancies, with clear-cell RCC (ccRCC) comprising 70–80% of cases as the most prevalent subtype [192, 193]. An in-depth analysis of ccRCC pathogenesis holds significant importance for clinical diagnosis and treatment. Glycosylation plays critical roles in ccRCC tumorigenesis and progression, where diverse glycosyltransferases mediate modifications that dysregulate signaling pathways [194, 195]. There are some glycosylation-related genes and proteins in ccRCC listed in Table 2.

**Table 2.** Glycosylation-related genes/proteins and their roles in RCC^a^.

Gene/protein	Glycosylation	Expression	Functions	Impact	Ref.
FUT3	α1,3-Fucose/LeX	Upregulation	Enhances the invasion and metastasis ability of tumor cells	Promotion	[196, 197]
ST6Gal-I	2,6-Galactose	Upregulation	Promotes the survival and migration of tumor cells and trigger immune escape	Promotion	[198, 199]
ST3Gal-I	α2,3-Galactose	Upregulation	High expression of ST3GAL-1 is associated with reduced OS (*P* = 0.013) and DFS (*P *= 0.004)	Promotion	[200]
ST3Gal-IV	α2,3-Galactose	Downregulation	Weakened tumor suppression	Suppression	[201]
ST84sia4	α2,8-Galactose	Upregulation	Promotes the proliferation and metastasis of ccRCC	Promotion	[202]
GnT-Ⅲ/IV	B1,6-Branching GlcNAc	Downregulation	GnT III and GnT IV activities decreased consistently in RCC	Promotion	[203]
GALNT2	*O*-GalNAc	Upregulation	Promotes the proliferation of tumor cells	Promotion	[204]
ST6GalNAc-I	STn antigen	Upregulation	Associated with tumor aggressiveness and poor prognosis of non-metastatic ccRCC	Suppression	[199]
GnT-III	Bisecting GlcNAc	Downregulation	Positively correlated with the TNM staging and metastasis of ccRCC	Promotion	[205]
RAGE/HMGB1	AGEs	Upregulation	HMGB1 activates the ERK1/2 signaling pathway via RAGE, promoting tumor growth and metastasis.	Promotion	[206]
CD147	*N*-Glycosylation	Upregulation	Promotes the activity of matrix metalloproteinases and enhance tumor invasion and angiogenesis	Promotion	[207]
Galectin-1	Galactin-binding glycan	Upregulation	Promotes the immunosuppressive microenvironment and inhibit T cell activity	Promotion	[208]
PGK1	*O*-GlcNAc	Upregulation	Promotes the Warburg effect in ccRCC	Promotion	[209, 210]

^a^OS, Overall survival; DFS, disease-free survival; AGEs, advanced glycation end products; TNM, tumor node metastasis classification.

Studies have demonstrated that proximal tubules of normal renal tissues are rich in biantennary *N*-GlcNAc and multi-antennary *N*-glycans with multiple fucose residues. However, these glycan structures are absent in ccRCC tissues, accompanied by the abnormal expression of tri-antennary or tetra-antennary *N*-glycans, as well as aberrations in high-mannose, sialylation, and fucosylation patterns [211, 212]. In the regulation of fucosylation, α1,3/α1,4-fucosyltransferase FUT3 is highly expressed in ccRCC [212]. FUT3 promotes invasion and immune escape by inducing EMT and enhancing tumor–macrophage communication [196]. Its high expression is correlated with shortened overall survival and recurrence-free survival in patients [213, 197]. Sialylation modifications drive tumor progression through multiple sialyltransferases (STs) [214, 215]. ST6Gal-I modifies Fas receptor via α2,6-sialylation to inhibit the apoptotic signaling pathway, enabling renal tumor cells to evade immune surveillance [198, 216]. Concurrently, miR-193a-3p and miR-224 suppress ST3Gal-IV via the PI3K/AKT pathway, altering sialylation patterns on tumor glycoproteins to enhance proliferation and invasion [201]. The long non-coding RNA HOTAIR acts as acompeting endogenous RNAs, upregulating ST8SIA-IV by sponging miR-124 to modulate sialylation and drive RCC progression [202]. *N*-Acetylglucosaminyltransferase V (GnT-V) catalyzes the synthesis of β1–6-branched GlcNAc on β1-integrin *N*-glycans, regulating the internalization and recycling of β1-integrin to influence the malignancy of renal cancer cells [217, 218].

Aberrant *O*-glycosylation modifications play a critical role in the oncogenesis and progression of ccRCC [219]. In the *O*-GalNAc pathway, truncated *O*-glycan Tn antigen (GalNAcα1-*O*-Ser/Thr) and its sialylated product STn antigen (Neu5Acα2–6GalNAcα1-*O*-Ser/Thr) exhibit abnormally high expression in cancer tissues [220, 221]. Specifically, the glycosyltransferase GALNT6 significantly enhances the proliferation, migration, and lung metastasis abilities of ccRCC cells by catalyzing the synthesis of Tn antigen in ccRCC [222]. ST6GalNAc-I, as a rate-limiting enzyme for STn antigen synthesis, is highly expressed in ccRCC [223]. This not only enhances the adhesion of tumor cells to the extracellular matrix by catalyzing the sialylation of mucins such as MUC5AC, but also mediates tumor immune escape by inducing the expression of sTn antigen, thereby promoting cancer cell proliferation and affecting the prognosis of patients [224]. In addition, other members of the mucin family also play important roles in glycosylation regulation of ccRCC. MUC1 is upregulated in ccRCC under the regulation of hypoxia-inducible factor HIF-1α, driving cancer cell invasion through promoting EMT [225]. MUC3A and MUC13 overexpression correlates with higher Fuhrman grade and may be PKC-regulated, suggesting therapeutic potential [226–228]. Notably, glycosylation defects in the cell surface laminin receptor dystroglycan (DG) disrupt cell–matrix interactions and activate pro-proliferative signaling pathways, thereby enhancing the invasive and proliferative capacities of ccRCC cells [229]. Additionally, *O*-GlcNAcylation catalyzed by OGT, is overexpressed in ccRCC and linked to tumor growth, invasion, metabolism, therapy resistance, and immune evasion [230, 231]. Mechanistically, OGT regulates *O*-GlcNAc modification of YTHDF1/YY1 to affect proliferation [230]. Its overexpression in ccRCC promotes cancer cell proliferation by inhibiting apoptosis and accelerating the cell cycle [232]. Moreover, OGT inhibits the degradation of HIF-2α by the ubiquitin–proteasome system, upregulates the expression of HIF-2α and its downstream target genes, and promotes tumor progression [233]. Thus, glycosylation modifications deeply participate in the initiation, invasion, metastasis, and immune escape of ccRCC through multidimensional mechanisms.

## Glycosylation as novel biomarkers and therapeutic targets in kidney diseases

Glycosylation modifications are closely linked to multiple kidney diseases, offering potential for precision diagnosis and targeted therapy. For instance, in hereditary kidney diseases, dysregulation of the *N*-glycosylation-dependent degradation pathway regulating ion channel protein stability revealed new treatment targets [234]. In renal fibrosis, targeting *N*-linked glycosylation of the type II TGF-β receptor (TβRII) blocks fibrogenic signaling [235]. In pMN, the aberrant *N*-glycosylation of pathogenic antibody IgG4 represents an emerging therapeutic opportunity [144]. Current research on glycosylation in kidney diseases is focused on IgAN, DKD, LN, RCC, and AKI. Extensive clinical and basic research has revealed disease-specific glycosylation profiles and established their pathological implications, with some key glycosylation biomarkers in kidney diseases summarized in Table 3. This section summarizes research advances in glycosylation across these conditions, evaluating their potential with regard to both biomarkers and therapeutic targets. By integrating glycobiology perspectives, these findings aim to catalyze precision medicine approaches for kidney diseases.

**Table 3.** Some potential biomarkers of glycosylation in kidney diseases.

Diseases	Biomarker	Sample	Method	Analyte	Ref.
IgAN	The sialylation level in the HR of IgA1	Human serum IgA HR	UHPLC-QqQ-MS/MS	*O*-Glycopeptide	[236]
	Gd-IgA1-specific antibodies	Human serum Gd-IgA1, Gd-IgA1-specific IgG, Gd-IgA1-specific IgA	Lectin ELISA	Protein	[237]
	The amount of GalNAc and galactose in the plasma IgA1 HR	Human plasma IgA1 HR	LC-MS/MS	*O*-Glycan	[238]
	The *N*-glycosylation profile of Umod	Human Umod	EThcD-sceHCD- MS/MS	*N*-Glycopeptide	[239]
DKD	Siaα2–6Gal/GalNAc, GlcNAc oligomers (2–4 units)	Human urine	Lectin microarray	Glycopeptide	[240]
	Urinary fetuin-A	Human urine	Lectin microarray, SSA-lectin affinity chromatography, LC-MS/MS	Glycoprotein	[241]
	AGEs	Human serum and plasma	LC-MS/MS	Urinary AGE compounds	[242]
LN	Man	Human kidney tissue	MALDI-MSI	*N*-Glycan	[243]
	*N*-Glycome, LacCer	Human serum and urine	MALDI-MSI	*N*-Glycan	[244]
	Levels of bisecting GlcNAc, sialylation, galactosylation, and fucosylation glycans of IgG	Human plasma IgG	HILIC-UPLC	*N*-Glycan	[245]
pMN	Galactose-deficient IgG4	Human serum IgG4	Nano-LC-MS, MALDI-MS	*N*-Glycopeptide, *N*-glycan	[246]
RCC	IgG galactosylation (Gal-ratio)	Human serum IgG	MS	*N*-Glycan	[247]
ccRCC	Prothrombin A2G2S glycan motif, immunoglobulin J chain FA2G2S2 motif, clusterin A2G2 motif, complement component C8A A2G2S2 motif, apolipoprotein M glycopeptide with non-fucosylated and non-sialylated hybrid-type glycan	Human plasma	LC-MS	Glycopeptide	[248]

**Abbreviations:** UHPLC-QqQ-MS/MS, Ultra high-performance liquid chromatography-triple quadrupole tandem mass spectrometry; ELISA, enzyme-linked immunosorbent assay; EThcD-MS/MS, electron-transfer/higher-energy collision dissociation tandem MS; AGEs, advanced glycation end products; MALDI-MSI, matrix-assisted laser desorption/ionization mass spectrometry imaging; LacCer, lactosylceramide; nano-LC-MS, nanoflow liquid chromatography-MS.

### IgA nephropathy

Emerging evidence highlights the diagnostic and therapeutic potential of targeting glycosylation in kidney diseases, with notable advancements in IgAN. Patients with IgAN exhibit significant *O*-glycosylation abnormalities in the HR of plasma IgA1, primarily characterized by reduced sialylation, galactosylation, and GalNAc levels [236]. The levels of Gd-IgA1 in serum or cell supernatants are significantly higher in IgAN patients than in healthy controls and those with non-IgAN kidney diseases, establishing Gd-IgA1 as a reliable diagnostic biomarker for IgAN [249]. Importantly, combined detection of blood GalNAc-Gal-IgA panels has been proven to have superior diagnostic efficacy compared to single markers or GalNAc-Gal combinations. Additionally, anti-Gd-IgA1 IgG antibodies demonstrate high sensitivity (89%) and specificity (92%) for IgAN diagnosis, and are closely associated with disease progression and prognosis [237]. Beyond *O*-glycosylation, Dotz *et al*. identified *N*-glycosylation signatures of IgA1 and IgA2 via LC-MS that exhibit higher diagnostic efficacy than *O*-glycosylation and are more closely correlated with deteriorating renal function [250]. Similarly, the *N*-glycosylation level of Umod is significantly reduced in IgAN patients, with modifications at the N396 site serving as a non-invasive diagnostic indicator [239]. FUT8 is an enzyme that catalyzes core fucosylation. Further expanding the biomarker spectrum, serum FUT8 activity positively correlates with tubulointerstitial damage, inflammation, and fibrosis severity, suggesting its utility as a biomarker/therapeutic target [217, 251]. Additionally, serum micro-RNAs (miRNAs) (e.g. let-7b and miR-148b) regulating the C1GALT1/Cosmc pathway and affecting IgA1 *O*-glycosylation serve as combined diagnostic markers [252].

Therapeutically, microbial protease therapy (e.g. recombinant IgA1 proteases) has successfully reduced glomerular IgA deposition and fibrosis in animal models and is now under evaluation in human trials [253–255]. Strategies such as developing glycan drugs targeting Gd-IgA1, inhibiting abnormal glycosylation enzyme activity, or eliminating Gd-IgA1-producing cells may serve as effective means for early intervention [256]. Studies have shown that miRNA inhibitors can promote the production of normal galactosylated IgA1 by upregulating C1GALT1 mRNA expression [256, 257]. Hydroxychloroquine, by inhibiting the TLR7/9 pathway, reduces the deposition of abnormally glycosylated IgA in the glomeruli, providing multifaceted strategies for precision therapy in IgAN [258]. Overall, these findings provide insights into the application of glycosylation modifications in the diagnosis and treatment of IgAN, and lay a multi-dimensional scientific foundation for the development of precision diagnosis and treatment strategies.

### Diabetic kidney disease

In diabetic kidney disease (DKD), glycosylation regulates disease progression through complex regulatory networks, with recent studies highlighting the significant value of *N*-glycosylation and *O*-glycosylation in both the diagnosis and mechanistic understanding of DKD. Regarding *N*-glycosylation's diagnostic value, plasma *N*-glycome analysis showed that the 2,6-sialylation of triantennary glycan A3E is strongly positively correlated with DKD risk, providing new directions for risk prediction and treatment monitoring [259]. Similarly, in urinary glycoproteomics, Guo *et al*. identified six differentially expressed *N*-glycoproteins, specifically highlighting that α-1-antitrypsin (SERPINA1) and ceruloplasmin serve as markers for distinguishing microalbuminuria from normoalbuminuria [260]. Inoue *et al*. found that urinary fetuin-A excretion is significantly increased in DKD patients, suggesting its potential as a biomarker for predicting the progression of type 2 DKD [241]. Complementing these, serum levels of advanced glycation end products (AGEs) are positively correlated with DKD progression, and combined testing can improve early diagnostic sensitivity [261]. Beyond diagnosis, *N*-glycosylation pathways also offer therapeutic targets. Receptor for avanced glycation end products (RAGE) antagonists can directly intervene in disease progression by inhibiting AGE-mediated inflammatory and fibrotic pathways [262]. In addition, ENTPD5 has emerged as a potential therapeutic target for intervening in DKD progression due to its ability to dynamically regulate ER *N*-glycosylation rates and alleviate ER stress [263]. Parallel to *N*-glycosylation, *O*-glycosylation plays a crucial pathogenic role in DKD. Several studies have demonstrated that OGT expression is significantly upregulated in renal tissues. Degrell *et al*. observed significantly elevated levels of *O*-GlcNAcylated proteins in DKD patient tissues versus controls [264]. Consistently, animal studies showed that *O*-GlcNAcylation, GFAT, and OGT protein expression were higher in the renal cortex and positively correlated with proteinuria [127]. Notably, Yu *et al*. analyzed *O*-glycans in the apical proximal tubule cells of rats with different levels of nephropathy and proteinuria and found that fucosylation levels were elevated in the CKD and the diabetes group, and the abundance of specific fucosylated *O*-mannans increased in the severe proteinuria group [265]. Collectively, these findings underscore the translational significance of both *N-* and *O*-glycosylation patterns in early disease diagnosis, progression assessment, and the development of targeted interventions.

**Figure 6.**
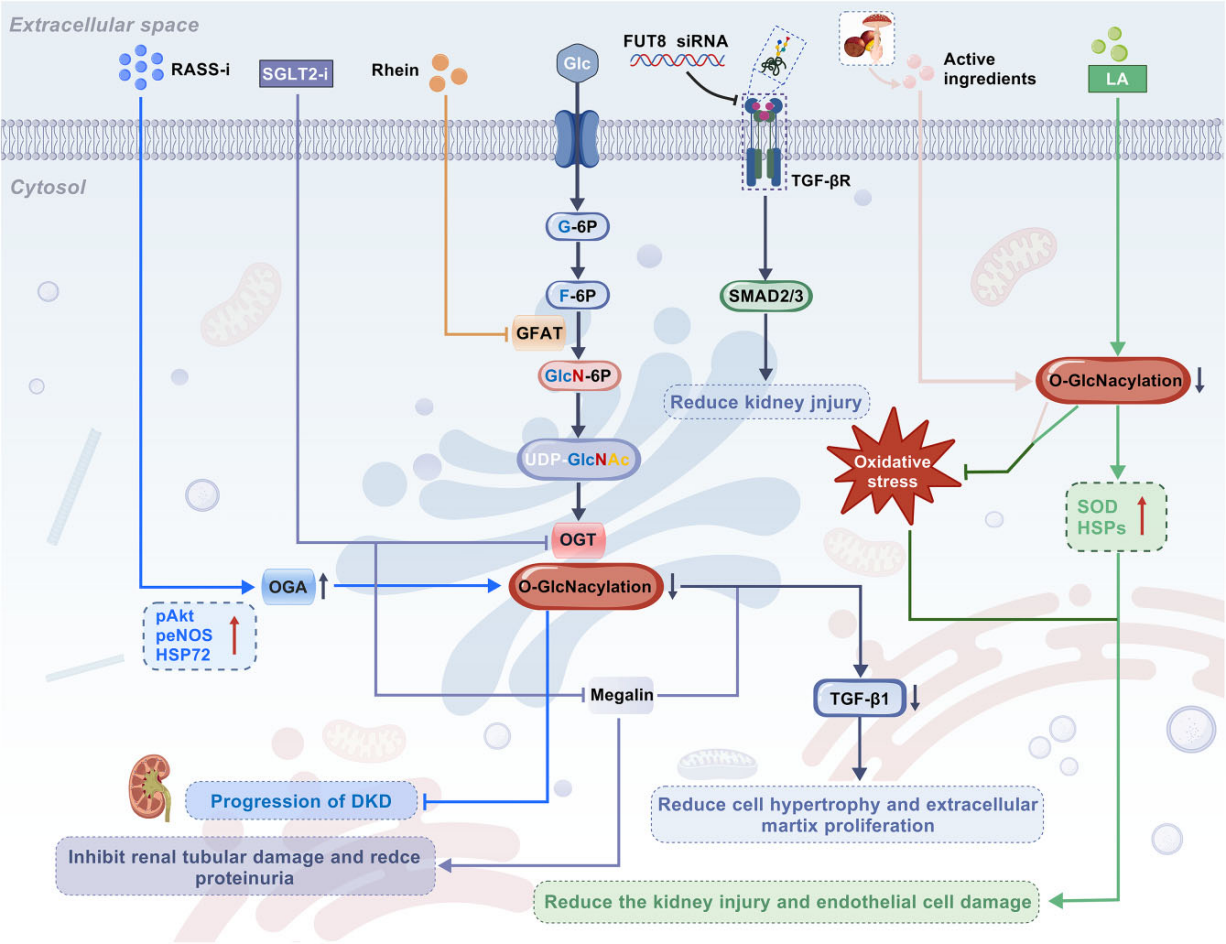
Mechanisms of multiple drugs inhibiting *O*-GlcNAcylation to alleviate kidney damage. “↑” Indicates activation, stimulation, or promotion, whereas “⊥” indicates inhibition, suppression, or decrease. Abbreviations: RAASi, activates the renin-angiotensin-aldosterone system inhibitor; SGLT-2i, sodium–glucose cotransporter-2 inhibitors; Glc, glucose; LA, α-lipoic acid; G-6P, glucose-6-phosphate; F-6P, fructose-6-phosphate; TGF-βR, transforming growth factor-β receptor; HSP72, heat shock protein 72; OGA, *O*-GlcNAcase; GFAT, glucosamino-fructose aminotransferase; GlcN-6P, glucosamine 6-phosphate; OGT, O-GlcNAc transferase; peNOS, phosphorylated endothelial nitric oxide synthase; pAKT, phosphorylated protein kinase B; SOD, CuZn-superoxide dismutase; HSPs, heat shock proteins. The figure was created with BioGDP (https://www.biogdp.com).

Inhibiting aberrant *O*-GlcNAcylation activation mitigates kidney injury and slows the progression of DKD to ESRD [266, 267]. Studies have shown that first-line drugs such as renin–angiotensin–aldosterone system (RAAS) inhibitors and sodium–glucose cotransporter-2 (SGLT2) inhibitors (e.g. dapagliflozin, empagliflozin) can exert renoprotective effects by targeting and regulating protein *O*-GlcNAc modifications. For example, RAAS inhibitors inhibit the abnormal elevation of *O*-GlcNAcylation by increasing *O*-GlcNAc enzymes and improve Akt/eNOS phosphorylation by restoring the vascular function, upregulating HSP72 expression, and thereby slowing the progression of DKD [122] (Fig. 6). Similarly, SGLT2 inhibitor dapagliflozin suppresses high glucose-induced aberrant *O*-GlcNAcylation by reducing OGT levels, which decreases renal tubular injury markers and connective tissue growth factor expression to alleviate tubular hypoxia [267]. Furthermore, empagliflozin inhibits *O*-GlcNAcylation of the proximal tubular receptor megalin, accelerating its internalization to block abnormal protein endocytosis and reduce proximal tubular protein overload, mitochondrial dysfunction, renal oxidative stress, and tubulointerstitial fibrosis [268] (Fig. 6).

Beyond pharmaceuticals, nutraceuticals show parallel *O*-GlcNAc-modulating effects. For instance, dietary α-lipoic acid (LA) supplementation reduces oxidative stress markers in the renal cortex of streptozotocin-induced diabetic rats, effectively preventing early glomerular injury [269]. In addition, in diabetic rat models, LA treatment restores CuZn-superoxide dismutase (SOD) and catalase activity, activates nuclear respiratory factor 2 (Nrf2)-mediated antioxidant gene expression by reducing *O*-GlcNAcylation of nuclear factors, and downregulates *O*-GlcNAc modifications of HSP90, HSP70, and MAPK signaling proteins. These coordinated effects enhance cellular resilience against diabetic oxidative damage [270–272] (Fig. 6). Likewise, some natural compounds and foods exhibit parallel renoprotective mechanisms. Rhein, a bioactive component from rhubarb, inhibits GFAT activity, reduces UDP-GlcNAc levels, and suppresses HBP/*O*-GlcNAc signaling in mesangial cells, thereby attenuating TGF-β1 expression, mesangial hypertrophy, and ECM proliferation [273] (Fig. 6). Similarly, mushroom and chestnut extracts reduce *O*-GlcNAcylation levels in diabetic models, mitigating oxidative stress and renal glucotoxicity [274] (Fig. 6). In addition to the potential therapeutic targets mentioned above, multiple molecular targets and intervention strategies have shown potential value in the treatment of kidney diseases. In DKD mouse models, studies have found that FUT8 small interfering RNA (siRNA) reduces the levels of renal tubular injury markers such as connective tissue growth factor and alleviates renal injury by inhibiting core fucosylation of TGF-β receptors and the activation of the Smad2/3 pathway [275–277] (Fig. 6). Collectively, these studies show that existing drugs, natural components, and novel molecules improve renal injury and delay renal fibrosis through inhibition of *O*-GlcNAcylation or related glycosylation pathways, offering potential optimization directions for clinical application.

### Lupus nephritis

In diagnostic and therapeutic applications, research reveals the value of glycosylation in lupus nephritis (LN). First, tissue-specific glycan alterations serve as robust diagnostic and prognostic markers. For example, Alves *et al*. found that mannose-type glycans in renal tissues of LN patients were abnormal, with 93% specificity in predicting CKD, positioning them as robust diagnostic and prognostic markers for LN [243]. Furthermore, neutrophil gelatinase-associated lipocalin, a glycoprotein upregulated during kidney injury, shows urinary levels that correlate more strongly with renal activity than anti-double strand (ds) DNA antibodies in LN [278]. Notably, Wolf *et al*. uncovered sex-specific urinary biomarkers, including lactosylceramide (LacCer) and *N*-glycans, with males showing more pronounced LacCer elevation linked to severe renal injury [244]. Secondly, studies highlight the critical pathophysiological role of IgG glycosylation. Research indicates that fucosylation of IgG can damage podocytes, whereas galactosylation has a protective effect [155, 156]. Further supporting this, Liou *et al*. further demonstrated that the sialic acid ratio of IgG anti-dsDNA antibodies inversely correlates with LN severity, with desialylated forms exacerbating inflammation and sialylated variants mitigating proteinuria [279]. Building on these mechanistic insights into glycosylation (particularly IgG glycosylation) in LN, glycosylation-targeted therapeutic strategies show clear promise. For example, disrupting IgG glycan structures blocks the podocyte injury pathway [280], fucosyltransferases inhibitors enable precise regulation of glycosylation levels, and enhancing antibody sialylation alleviates renal injury. Moving forward, translating these findings to clinical practice will require overcoming technical barriers in glycomic analysis, validating biomarker efficacy across diverse populations, and integrating sex-specific insights into personalized treatment pathways, particularly those gleaned from biomarker studies.

### Renal cell carcinoma

Renal cell carcinoma (RCC), the most common genitourinary cancer, accounts for 90% of malignant kidney tumors, with the highly malignant ccRCC subtype being predominant [192, 281]. Its complex histopathology contributes to late diagnosis, chemoresistance, and high mortality (30%–40%), driving the search for improved diagnostic tools [238, 282]. In recent years, an increasing number of studies have revealed the molecular regulatory mechanisms in ccRCC, providing important evidence for the development of its diagnostic markers [283]. Notably, alterations in specific glycosylation patterns also exhibit potential value in the clinical application of ccRCC. MS imaging analyses comparing normal renal tissues and ccRCC tissues showed significant loss of biantennary *N*-GlcNAc and fucose-rich multiantennary *N*-glycans in tumors, accompanied by tumor-specific tri-/tetra-antennary glycans with variable sialylation/fucosylation patterns, suggesting their potential as biomarkers [211]. Complementing this, core fucosylation of cluster proteins is markedly upregulated in ccRCC. Building on tissue findings, plasma analyses offer dynamic monitoring potential. Gbormittah *et al*. identified elevated levels of core-fucosylated diantennary (FA2G2S2) and triantennary (A3G3S2) glycans in post-nephrectomy plasma, suggesting dynamic glycan changes as postoperative monitoring biomarkers [284]. Similarly, Gbormittah *et al*. demonstrated significant N374 glycoform heterogeneity in plasma clusterin before and after radical nephrectomy, providing insights for therapeutic targeting. Similarly, serum-based approaches further underscore glycosylation's diagnostic potential [284]. Hatakeyama *et al*. identified statistically elevated P40 and P43 serum glycans as robust ccRCC diagnostic markers [285]. Leveraging IgG glycosylation, Ren *et al*. constructed a galactosylation-based Gal ratio model (area under the curve > 0.8) distinguishing early-stage RCC from non-cancer controls, enabling non-invasive screening [247]. In another study, Serie *et al*. screened five glycopeptides in plasma, including prothrombin (A2G2S glycoform) and apolipoprotein M, which can predict postoperative progression-free survival, with a multivariate model achieving a hazard ratio of 11.96 (*P *< 0.0001), outperforming single-marker approaches [248].

Urine-based analyses represent a critical approach in ccRCC research. For example, Santorelli *et al*. identified distinct proteomic alterations in ccRCC urine via LC-MS/MS, including upregulated CD97 and coagulation factor C homologs alongside downregulated APOB, FINC, and CERU. Notably, these expression trends correlated with tumor stage [286]. Beyond specific proteins, dysregulation within the glycosylation machinery holds significant clinical value. Specifically, in the fucosyltransferase family, overexpression of FUT3 can serve as a molecular marker for poor prognosis, while the high differential expression of FUT11 indicates tumor progression potential, rendering both promising candidates for risk stratification models [213, 287]. Similarly, in the sialyltransferase family, high expression of ST3Gal-I and ST6Gal-I is significantly associated with reduced patient survival. Furthermore, increased levels of sialylated Lewis antigens can serve as predictive indicators of lymph node metastasis, providing valuable evidence for preoperative metastasis assessment [198, 216, 200, 288]. Therapeutically, glycosylation inhibitors targeting glycosyltransferases have shown potential application prospects. *O*-Glycosylation also plays an important role in the diagnosis and treatment of ccRCC. Elevated *O*-GlcNAcylation and OGT expression act as oncogenic factors in renal cancer development, positioning OGT as a potential therapeutic target for renal cancer [289]. *N*-Acetylgalactosaminyltransferases (GALNTs), which initiate *O*-GlcNAcylation, play a pivotal role in cancer progression [290–292]. Notably, the sTn antigen is a truncated *O*-glycan structure that is significantly up-regulated in ccRCC and has become a signature molecule of tumor-associated glycan antigen [293]. ST6GalNAc-1 influences tumor activity by regulating Tn and sTn antigens, and its high expression is associated with poor survival prognosis in non-metastatic patients [294, 295]. These findings suggest that ST6GalNAc-1 can serve as a potential therapeutic target. GALNT6, overexpressed in ccRCC, drives Tn antigen synthesis and promotes tumor proliferation through aberrant *O*-glycosylation, underscoring its potential as a therapeutic target [222]. Similarly, elevated expression of GALNT2 in ccRCC correlates with enhanced tumor growth and worse patient outcome [204]. Mechanistically, miR-139–5p targets GALNT2 to suppress large tumor suppressor kinase 2 (LATS2) activation, thereby driving ccRCC proliferation, revealing the miR-139–5p–GALNT2–LATS2 axis as a novel therapeutic opportunity [204].

### Acute kidney injury

Acute kidney injury (AKI), characterized by rapid renal function decline, manifests as abrupt loss of kidney function within a short period. Its progression can lead to irreversible damage to nephrons and is closely associated with the development of CKD [296]. Recent research highlights glycosylation's protective role in AKI, revealing promising intervention strategies. Specifically, *O*-GlcNAc modification alleviates renal injury through multi-dimensional mechanisms. In contrast-induced AKI models, enhanced *O*-GlcNAcylation levels via glucosamine promote phosphorylation of Akt, upregulate the anti-apoptotic protein Bcl-2, downregulate Bax, and suppress oxidative stress and apoptosis [297]. Remote ischemic preconditioning similarly exerts protective effects by increasing renal *O*-GlcNAcylation [298]. In ischemic AKI, GlcN supplementation enhances *O*-GlcNAc modification of Sp1, activating GRP78/HIF-1α and improving SGLT function to alleviate hypoxic injury [109, 299]. Additionally, GALNT3 is downregulated in ischemic AKI and cisplatin nephrotoxicity, where it activates survival signals by promoting *O*-glycosylation of the epidermal growth factor receptor to inhibit tubular apoptosis [300]. Furthermore, fucosylation contributes significantly to AKI protection. Fucosylated Tamm-Horsfall protein inhibits complement lectin pathway activation via enhanced collectin-11 binding, positioning fucose as a therapeutic target for AKI [301]. Collectively, these studies provide novel insights for improving the prognosis of AKI patients and blocking their progression to CKD.

## Conclusion

Glycosylation, a pivotal form of protein PTM, dynamically regulates protein function, intercellular signaling, and metabolic networks to drive the pathogenesis and progression of kidney diseases. Aberrant glycosylation has been identified across diverse nephropathies, including IgAN, DKD, RCC, ADPKD, and AKI etc. These glycosylation alterations impact multiple facets of renal cell biology, intercellular interactions, and immune responses, thereby modulating disease trajectory. In recent years, glycosylation has emerged as a promising frontier in the diagnosis and treatment of renal diseases, but its clinical translation remains hindered by multifaceted challenges. The complexity of glycan structures and the microheterogeneity of low-abundance pathological glycoproteins fundamentally restrict the sensitivity of detection and structural analysis, while the spatiotemporal dynamics and tissue specificity of glycosylation remain poorly understood. Most clinical studies are limited by small sample sizes and animal models fail to accurately recapitulate human glycosylation profiles. Technical bottlenecks further impede progress, including inadequate coverage for glycan isomer detection, absence of standardized sample processing protocols, and computational barriers in glycopeptide spectrum annotation and multi-omics integration—all of which collectively undermine biomarker validation and point-of-care application.

As the molecular mechanisms of glycosylation in kidney diseases are increasingly unraveled, future research should focus on innovations in automated microsampling, high-resolution isomer separation techniques, and artificial inteligence-driven predictive algorithms. Through the integration of single-cell histology, artificial intelligence, and next-generation detection platforms, it will be possible to elucidate how glycosylation heterogeneity regulates renal cell function spatiotemporally, screen disease-specific glycosylation signatures, and develop small-molecule inhibitors targeting glycosyltransferases, glycosylation remodeling therapies, and non-invasive biomarkers. Leveraging organoid models and interdisciplinary collaboration will accelerate the clinical translation of precision diagnostic strategies, ultimately enabling early intervention and personalized therapeutic regimens of renal diseases.
